# Identification of the BRD1 interaction network and its impact on mental disorder risk

**DOI:** 10.1186/s13073-016-0308-x

**Published:** 2016-05-03

**Authors:** Tue Fryland, Jane H. Christensen, Jonatan Pallesen, Manuel Mattheisen, Johan Palmfeldt, Mads Bak, Jakob Grove, Ditte Demontis, Jenny Blechingberg, Hong Sain Ooi, Mette Nyegaard, Mads E. Hauberg, Niels Tommerup, Niels Gregersen, Ole Mors, Thomas J. Corydon, Anders L. Nielsen, Anders D. Børglum

**Affiliations:** Department of Biomedicine, Aarhus University, Building 1242, Bartholins Allé 6, 8000 Aarhus C, Denmark; iPSYCH, The Lundbeck Foundation Initiative for Integrative Psychiatric Research, 8000 Aarhus C, Denmark; iSEQ, Centre for Integrative Sequencing, Aarhus University, 8000 Aarhus C, Denmark; Research Unit for Molecular Medicine, Aarhus University Hospital, 8200 Skejby, Denmark; Wilhelm Johannsen Centre for Functional Genome Research, Department of Cellular and Molecular Medicine, University of Copenhagen, 2200 Copenhagen N, Denmark; Bioinformatics Research Centre (BiRC, Aarhus University, 8000 Aarhus C, Denmark; Research Department P, Aarhus University Hospital, 8240 Risskov, Denmark

**Keywords:** Functional genomics, Mental disorders, Schizophrenia, Interactome, Regulome, Proteomics, Chip-seq

## Abstract

**Background:**

The bromodomain containing 1 (BRD1) gene has been implicated with transcriptional regulation, brain development, and susceptibility to schizophrenia and bipolar disorder. To advance the understanding of BRD1 and its role in mental disorders, we characterized the protein and chromatin interactions of the BRD1 isoforms, BRD1-S and BRD1-L.

**Methods:**

Stable human cell lines expressing epitope tagged BRD1-S and BRD1-L were generated and used as discovery systems for identifying protein and chromatin interactions. Protein-protein interactions were identified using co-immunoprecipitation followed by mass spectrometry and chromatin interactions were identified using chromatin immunoprecipitation followed by next generation sequencing. Gene expression profiles and differentially expressed genes were identified after upregulating and downregulating BRD1 expression using microarrays. The presented functional molecular data were integrated with human genomic and transcriptomic data using available GWAS, exome-sequencing datasets as well as spatiotemporal transcriptomic datasets from the human brain.

**Results:**

We present several novel protein interactions of BRD1, including isoform-specific interactions as well as proteins previously implicated with mental disorders. By BRD1-S and BRD1-L chromatin immunoprecipitation followed by next generation sequencing we identified binding to promoter regions of 1540 and 823 genes, respectively, and showed correlation between BRD1-S and BRD1-L binding and regulation of gene expression. The identified BRD1 interaction network was found to be predominantly co-expressed with BRD1 mRNA in the human brain and enriched for pathways involved in gene expression and brain function. By interrogation of large datasets from genome-wide association studies, we further demonstrate that the BRD1 interaction network is enriched for schizophrenia risk.

**Conclusion:**

Our results show that BRD1 interacts with chromatin remodeling proteins, e.g. PBRM1, as well as histone modifiers, e.g. MYST2 and SUV420H1. We find that BRD1 primarily binds in close proximity to transcription start sites and regulates expression of numerous genes, many of which are involved with brain development and susceptibility to mental disorders. Our findings indicate that BRD1 acts as a regulatory hub in a comprehensive schizophrenia risk network which plays a role in many brain regions throughout life, implicating e.g. striatum, hippocampus, and amygdala at mid-fetal stages.

**Electronic supplementary material:**

The online version of this article (doi:10.1186/s13073-016-0308-x) contains supplementary material, which is available to authorized users.

## Background

Genetic variations in the bromodomain containing 1 (BRD1) gene located on 22q13.33 have repeatedly been associated with both schizophrenia and bipolar disorder [1–3]. The BRD1 locus approached genome-wide significance (*P* = 3.31 × 10^–7^) in the most recent schizophrenia genome-wide association study (GWAS) by the Psychiatric Genomics Consortium using conventional statistical methods [4]. Moreover, the locus was found genome-wide significant using an Empirical Bayes statistical approach and predicted to be highly replicable [5]. Furthermore, in a schizophrenia GWAS meta-analysis (11,185 cases/10,768 controls) and a family-based replication study (3286 cases from 1811 families) the SNP rs138880 in the promoter region of BRD1 showed the overall most significant association [6].

BRD1 is essential for normal brain development and inactivation of both alleles of *Brd1* in mice leads to impaired neural tube closure [7]. Co-immunoprecipitation (co-IP) of epitope tagged and endogenous BRD1 and MYST2 from human K562 and HEK293 cells suggest that ING4, MEAF6, and MYST2 constitute the primary histone acetyltransferase complex of BRD1 [7]. Additionally, a focused promoter ChIP-on-chip (chromatin immunoprecipitation combined with microarray analysis) of co-expressed epitope tagged BRD1 and MYST2 in human K562 cells identified a large overlap in target genes between the two proteins suggesting a pivotal role of the BRD1/MYST2 complex in transcriptional regulation [7]. Equally, *Brd1*^–/–^ and *Myst2*^–/–^ knockout mouse embryos show severely decreased levels of the overall histone H3K14 acetylation, suggesting that the Brd1/Myst2 complex is responsible for the majority of histone H3K14 acetylation during embryonic development in mice [7, 8]. We have previously reported a divergence in immunohistochemical staining between the BRD1-S and BRD1-L isoforms in the brain [9] as well as differential transcriptional regulation of the *Brd1-S* and *Brd1-L* splice variants in prefrontal cortex and hippocampus following chronic restrained stress [10] and electroconvulsive seizures [11] in adult rats, indicating that BRD1 isoforms can perform separate functions dependent on the specific cell type and tissue. To gain more knowledge about the biological functions of BRD1 and how these might be involved in the pathogenesis of schizophrenia and related mental disorders, we sought in the present study to identify and analyze the BRD1 interaction network, encompassing BRD1-S and BRD1-L protein-protein interactions (PPIs) and chromatin interactions as well as genes being regulated upon up- or downregulation of BRD1. Moreover, we interrogated large GWAS datasets and found that the BRD1 interaction network is enriched for schizophrenia risk.

## Methods

### Cell work

The generation of cell lines stably expressing BRD1-S-V5 and BRD1-L-V5 have previously been described [9]. HEK293T cells (controls and stable BRD1-S-V5 and BRD1-L-V5 cell lines) were grown in DMEM medium (Invitrogen, San Diego, CA, USA) supplemented with 5 % fetal calf serum (FCS), 175 mg/L glutamine, 36 mg/L penicillin, and 60 mg/L streptomycine at 37 °C in 5 % CO_2_.

### Co-immunoprecipitation (Co-IP)

Preparation of cell extract was performed according to the two-step procedure described in [12]. Experiments were carried out in 10 cm or 15 cm petri dishes with 1 × 10^7^ cells or 2 × 10^7^ cells plated, respectively. 1 × 10^8^ cells were used for each immunoprecipitation (IP). Cells were counted using a Nucleocounter (ChemoMetec A/S, Alleroed, Denmark) and plated 24 h before harvested using 1 mL per 10 × 10^6^ cells hypotonic Triton X-100 lysis buffer (20 mM Tris–HCl [pH 7.4], 10 mM KaCl, 10 mM MgCl_2_, 2 mM EDTA, 10 % glycerol, 1 % Triton X-100, 2.5 mM β-glycerophosphate, 1 mM NaF, 1 mM DTT + protease inhibitors (Roche, Mannheim, Germany]) for 10 min on ice. Cell lysate was distributed to 15 mL tubes with 2 mL in each for sonication. DNA was fragmented by sonication (Bioruptor, settings: on 0.5, off 0.5) for 15 min at 6 °C. A total of 5 M NaCl was added to a final concentration of 420 mM, mixed and incubated on ice for 15 min after which the DNA fragmentation was repeated. Sonicated cell lysate was then cleared by centrifugation at maximum speed for 15 min and the supernatant was recovered for IP.

IP of V5 epitope tagged proteins was performed as follows: Anti-V5 and anti-HA antibody conjugated agarose beads (Sigma Aldrich, Steinheim, Germany) were washed twice in PBS before use and blocked in 1 % BSA. Cell lysates were pre-cleared for 30 min at 4 °C under rotation using 30 μL anti-HA antibody conjugated bead solution before collecting the supernatant (400 rpm for 5 min) for IP. IP was performed using 60 μL of the bead solution per 10 mL lysate. Beads were added to the pre-cleared supernatant and incubated at 4 °C overnight before they were collected at 400 rpm for 15 min. Beads were washed twice in 10 mL Triton X-100 buffer 250 (20 mM Tris–HCl [pH 7.4], 250 mM NaCl, 10 mM MgCl_2_, 2 mM EDTA, 10 % glycerol, 1 % Triton X-100, 2.5 mM β-glycerophosphate, 1 mM NaF, 1 mM DTT, protease inhibitors [Roche]) then moved to Protein LoBind tubes (Eppendorf, Hamburg, Germany) and then washed six times in Triton X-100 buffer 150 (20 mM Tris–HCl [pH 7.4], 150 mM NaCl, 10 mM MgCl_2_, 2 mM EDTA, 10 % glycerol, 1 % Triton X-100, 2.5 mM β-glycerophosphate,1 mM NaF, 1 mM DTT, protease inhibitors [Roche]). Proteins were eluted in 100 μL Elution buffer (HENG) (10 mM HEPES-KOH [pH 9.0], 1.5 mM MgCl2, 0.25 mM EDTA, 20 % glycerol, 250 mM KCl, 0.3 % NP40 + 0.5 mg/mL V5 peptide [Sigma], protease inhibitors [Roche]). Cell lysate was kept cold at all times, between 1 °C and 6 °C. The process was carried out in one continuous procedure without any freezing/thawing of the cell lysate. IP with anti-V5 antibody and IP with anti-HA antibody was performed four times with either extracts from HEK293T, stable BRD1-S-V5, or BRD1-L-V5 expressing cell lines, adding to a total of 24 IP samples prepared for mass spectrometry.

IP of endogenous PBRM1 was performed using the same procedure as described above. Protein A and G beads (GE Healthcare, Uppsala, Sweden) were washed twice in PBS and blocked in 1 % BSA (final concentration). Pre-clearing and IP was performed in a 50/50 μL mix of blocked protein A and G bead solutions. A total of 30 μL of protein A and G bead solution was used for preclearing of cell extracts and 60 μL of protein A and G bead solution was used for IP. Anti-PBRM1 antibody (Cat. no. A301-590A, Bethyl, Montgomery, AL, USA) was diluted in Triton X-100 buffer 150 according to antibody specifications and incubated with blocked protein A and G bead solution at 4 °C overnight. IP control reactions were performed in parallel by the same method but without the addition of antibody. Furthermore, elution was performed with 1 % SDS instead of the V5 peptide solution as described above.

### Sample preparation for mass spectrometry

Proteins eluted upon IP were precipitated in six volumes of acetone (–20 °C), incubated at –20 °C overnight, and centrifuged at 15,000 × g at 4 °C. The supernatant was cautiously removed and the protein pellet was carefully washed in ice-cold 90 % acetone, centrifuged at 15,000 g and air-dried. Disulfide bonds of the proteins were reduced in 90 μL buffer containing 50 mM tris-(2-carboxyethyl)phosphine (TCEP) (Sigma Aldrich, Steinheim, Germany) and 50 mM ammonium bicarbonate, and incubated at 60 °C for 10 min. The reduced cystein residues were blocked by 50 mM of the alkylation agent iodoacetamide (Sigma-Aldrich, Steinheim, Germany). Proteins were digested into peptides by addition of 1 μg trypsin (Trypsin Gold from Promega, Madison, WI, USA) in 100 μL 50 mM ammonium bicarbonate.

### Mass spectrometry analysis

The peptide mixtures were analyzed by nanoliquid chromatography (Easy nLC from Proxeon, Denmark) coupled to mass spectrometry (MS) (Thermo Fisher, LTQ-Orbitrap), with separation on a reverse phase column (75 μm, 100 mm, and 3.5 μm C18 particles) at a flow rate of 300 nL/min using a 100 min gradient (5–35 %) of acetonitrile in 0.4 % acetic acid. The MS detection constituted a full scan (m/z 400 − 2000) in Orbitrap (<3 ppm mass accuracy) followed by up to four data dependent MS/MS fragmentation scans using collision induced dissociation (CID).

### Database searches and MS statistics

The resulting MS files were processed essentially as previously described [13]. Briefly, Mascot version 2.2.04 (Matrix Science) was used for peptide identification and MaxQuant version 1.0.13.18 for protein identification and label-free quantification [14]. The MS data were searched against IPI protein database version 3.52 containing 73,928 sequences and the same number of reversed sequences for false discovery rate calculations (FDR). FDR was set to 0.01 for both identification of peptides and proteins. MS/MS mass tolerance was 5 ppm for peptide masses and 0.5 Da for fragment masses. Setting of trypsin digestion was cleavage at C-terminus of lysine and arginine except before proline and up to two missed cleavages were accepted. Carbamidomethylation at cysteine residues was set as fixed modification and oxidation of methionine was set as variable modification. Only peptides with a minimum length of six amino acid residues were accepted and at least two peptides (and one unique peptide) were required for protein identification.

### Proteomics data analysis

The combined spectral intensity of each individual protein was normalized to the mean intensity of all the proteins for each sample or control. Intensities below the detection limit were assigned the value of mean of the minimum intensities from across all datasets, and then normalized with the mean intensity of all values in the sample or control. Extracts from stable BRD1-S-V5 and BRD1-L-V5 expressing cells and from HEK293T cells were used in co-IP experiments. Each co-IP experiment was performed four times followed by LC-MS/MS analysis, using beads with anti-V5 conjugated antibody (IP:V5) or anti-HA conjugated antibody (IP:HA) and extracts from stable BRD1-S-V5, BRD1-L-V5 expressing cells, or HEK293T cells, yielding: (4 × IP:V5 4 × BRD1-S-V5, 4 × IP:HA BRD1-S-V5, 4 × IP:V5 BRD1-L-V5, 4 × IP:HA BRD1-L-V5, 4 × IP:V5 HEK293T, 4 × IP:HA HEK293T). Four criteria (1–4) were raised to systematically identify repeatedly co-immunoprecipitated (co-IPed) proteins by calculating the enrichment ratio (log_2_ (sample) – log_2_ (control)), where control experiment is IP without proper BRD1 in the sample or without antibody against the proper BRD1 construct. The four criteria were: (1) the enrichment ratio IP:V5 BRD1-(S or L)-V5 - IP:HA BRD1-(S or L)-V5 of a protein is > cutoff value; (2) the enrichment ratio IP:V5 BRD1-(S or L)-V5 - IP:V5 HEK293T of a protein is > cutoff value; (3) the enrichment ratio IP:V5 BRD1-(S or L)-V5 - IP:HA HEK293T of a protein is > cutoff value; (4) the enrichment ratio IP:V5 BRD1-(S or L)-V5 - the mean of all controls of a protein is > cutoff value. Both two- and threefold cutoffs were applied to sort the identified protein-protein interactions according to co-IP strength (Table 1).

**Table 1:** Summary of BRD1 PPIs identified by co-IP mass spectrometry

Co-IP MS/MS	Co-IP strength^a^	Protein	Uniprot ID	Gene name	Peptide P value^b^
BRD1-S	1	BRD1	O95696	Bromodomain-containing protein 1	<1 × 10^–10^
		ZC3H15	Q8WU90	Zinc finger CCCH domain-containing protein 15	<1 × 10^–10^
		DRG1	Q9Y295	Developmentally regulated GTP-binding protein 1	<1 × 10^–10^
		C1QBP	Q07021	Complement component 1 Q sub-component-binding protein	<1 × 10^–10^
		MYST2	O95251	K(lysine) acetyltransferase 7	<1 × 10^–10^
		SUV420H1	Q4FZB7	Suppressor of variegation 4-20 homolog 1	<1 × 10^–10^
		MEAF6	Q9HAF1	MYST/Esa1-associated factor 6	<1 × 10^–10^
		YWHAE	P62258	14-3-3 protein epsilon	5.1 × 10^–9^
		PBRM1	Q86U86	Polybromo-1	2.7 × 10^–3^
		ING4	Q9UNL4	Inhibitor of growth protein 4	4.2 × 10^–3^
		ING5	Q8WYH8	Inhibitor of growth protein 5	1.1 × 10^–2^
	2	NPM1	P06748	Nucleophosmin	<1 × 10^–10^
		YWHAZ	P63104	14-3-3 protein zeta/delta	<1 × 10^–10^
		KPNA1	P52294	Importin subunit alpha-1	<1 × 10^–10^
		KPNA2	P52292	Importin subunit alpha-2	<1 × 10^–10^
		CHD3/CHD4/CHD5^3^	Q14839/Q12873/Q8TDI0^3^	Chromodomain-helicase-DNA-binding protein 3/4/5^3^	<1 × 10^–10^
		YWHAB	P31946	14-3-3 protein beta/alpha	1.6 × 10^–7^
		NAP1L4	Q99733	Nucleosome assembly protein 1-like 4	1.6 × 10^–7^
		CLTC	Q00610	Clathrin heavy chain 1	2.7 × 10^–6^
		DNMT1	P26358	DNA (cytosine-5)-methyltransferase 1	1.6 × 10^–5^
		LYAR	Q9NX58	Cell growth-regulating nucleolar protein	2.1 × 10^–5^
		MRPS14	O60783	28S ribosomal protein S14	5.6 × 10^–4^
		EIF3I	Q13347	Eukaryotic translation initiation factor 3 subunit I	5.2 × 10^–3^
	3	PHB2	Q99623	Prohibitin-2	8.4 × 10^–7^
		NSUN2	Q08J23	NOP2/Sun RNA methyltransferase	7.3 × 10^–3^
BRD1-L	1	BRD1	O95696	Bromodomain-containing protein 1	<1 × 10^–10^
		YWHAE	P62258	14-3-3 protein epsilon	<1 × 10^–10^
		ZC3H15	Q8WU90	Zinc finger CCCH domain-containing protein 15	<1 × 10^–10^
		DRG1	Q9Y295	Developmentally regulated GTP-binding protein 1	<1 × 10^–10^
		YWHAG	P61981	14-3-3 protein gamma	<1 × 10^–10^
		MEAF6	Q9HAF1	MYST/Esa1-associated factor 6	8.5 × 10^–7^
		ING5	Q8WYH8	Inhibitor of growth protein 5	9.1 × 10^–4^
		PBRM1	Q86U86	Polybromo-1	1.3 × 10^–3^
		MRPS34	O60783	28S ribosomal protein S14	1.5 × 10^–3^
		ING4	Q9UNL4	Inhibitor of growth protein 4	6.2 × 10^–3^
		QSER1	Q2KHR3	Glutamine and serine-rich protein 1	6.4 × 10^–3^
	2	YWHAZ	P63104	14-3-3 protein zeta/delta	<1 × 10^–10^
		CLTC	Q00610	Clathrin heavy chain 1	9.8 × 10^–4^
	3	H3F3A/HIST1H3/HIST3H3^c^	P84243/P68431/Q16695^c^	Histone H3.3/Histone H3.1/Histone H3.1 t^c^	<1 × 10^–10^
		YWHAH	Q04917	14-3-3 protein eta	<1 × 10^–10^
		C1QBP	Q07021	Complement component 1 Q sub-component-binding protein	<1 × 10^–10^
		RPL22L1	Q6P5R6	60S ribosomal protein L22-like	<1 × 10^–10^

^a^Co-IP strength, 1: co-IPed 3–4 out of four repeated experiments and sample/control enrichment ratio >3 across four control experiments, 2: co-IPed 3–4 times out of four repeated experiments and sample/control enrichment ratio >2 across four control experiments, 3: co-IPed two out of four repeated experiments and sample/control enrichment ratio >3 across four control experiments (for further details see “Methods”)

^b^Co-IPed proteins were sorted by the peptide *P* value (MaxQuant software) within each of three settings for co-IP strength

^c^Identified peptides were unique to all listed proteins (encoded by more than one genetically related gene)

### Western blotting

Protein samples were prepared for SDS-PAGE using 5× loading buffer and 20× reducing agent (Fermentas, St. Leon-Rot, Germany) and then placed in a heating block for 5 min at 100 °C. Denatured proteins were loaded onto TGX or Tris-HCL 4–15 % linear gradient polyacrylamide gels (Bio-Rad Laboratories) and separated by electrophoresis at 100–120 V. Proteins were transferred to Hybond-P Membranes (GE Healthcare, Uppsala, Sweden) using a wet blotting apparatus (Bio-Rad Laboratories). Membranes were activated in 99 % alcohol for 20 s, washed in redistilled water for 1 min, and transferred to transfer buffer (Tris 50 mM Tris, 385 mM glycine, 7 mM SDS, 10 % Ethanol). Membranes were blocked for 1 h in blocking buffer (10 % skimmed milk powder, 1 % Tween 20, and PBS). Primary antibodies were: monoclonal anti-V5 antibody (Invitrogen, R960-25) diluted 1:2000 in wash buffer (0.05 % Skimmed milk powder, 0.005 % Tween 20, and PBS) and anti-BRD1 antibody (65/66 Sigma Genosys), previously described [9]. Secondary antibodies were: HRP conjugated anti-mouse IgG antibody (Dako Cytomation, P0447, Glostrup, Denmark) and HRP conjugated anti-rabbit IgG antibody (Dako Cytomation, P0448, Glostrup, Denmark). Dilutions were according to the manufacturer’s protocol. All incubations were performed in 5–10 mL wash buffer. Membranes were incubated with primary antibodies for 24 h at 4 °C, followed by 3× 5 min wash and for 1 h with secondary antibody at 4 °C and washed 5× 5 min with wash buffer. Immunoreactivity was detected using BM Chemoluminescence substrate (POD) (Roche Applied Science, Mannheim, Germany) according to the manufacturer’s protocol and the Image Reader LAS-3000 v2.2 program (Fujifilm, Minato (Tokio), Japan). Densitometric analysis was performed with the Multi Gauge v3.1 software (Fujifilm, Minato (Tokio), Japan).

### Immunofluorescence

HEK293T cells stably expressing BRD1-S and BRD1-L and untransfected HEK293T cells were cultured for 48 h in 10 cm^2^ slideflasks. Cells were fixed in 4 % freshly prepared paraformaldehyde, 0.1 % gluteraldehyde, 0.1 % Trition X-100 solution on ice for 30 min, followed by wash with PBS for 5 min. Slides were incubated with anti-V5 antibody (Invitrogen) for 1 h at 4 °C using a 1:200 dilution in PBS and washed 3× 5 min with PBS containing 0.1 % Triton X-100. Incubation with anti-mouse immunoglobulins/FITC F(ab’)2 (Dako, Glostrup, Denmark) was performed for 1 h at 4 °C using a 1:200 dilution in PBS supplemented with 5 % FCS. Cell nuclei were stained by Hoechst staining (Hoechst stain 1 μg/mL) for 10 min, slides were then washed 2× 5 min in PBS containing 1 % Triton X-100 and dried. Two drops of fluorescent mounting media were added to the fixed cells and stored in the dark at 4 °C until media were dried out and then viewed under a fluorescent microscope (Additional file 1).

### Chromatin immunoprecipitation (ChIP)

ChIP of V5 epitope proteins were carried out as described in [15] with a few adjustments. Eight million cells per reaction were seeded 24 h before use in 10 mL medium. Formaldehyde, to the final concentration of 1 %, was added directly to the cell medium, on a shaker. The cross-linking reaction was stopped after 10 min by addition of glycine to the final concentration of 0.125 M, on a shaker. Cells were carefully washed twice in 10 mL cold PBS, harvested in 1 mL ChIP dilution buffer (0.01 % SDS, 1.1 % Triton X-100, 1.2 mM EDTA, 16.7 mM Tris-HCL, 167 mM NaCl) and protease inhibitors (Complete mini, Roche, Mannheim, Germany) and sonicated on ice (Bioruptor, settings: on 0.5, off 1.0) for 15 min at 6 °C. Cell lysate was cleared by 2 min maximum centrifugation at 6 °C. Supernatant was collected and stored at –80 °C.

Immunoprecipitations were carried out using 20 μL of anti- V5 or anti- HA conjugated agarose beads (Sigma, A7345, A2095). First, the beads were washed twice in PBS and protease inhibitors (Roche, Mannheim, Germany) with intermediate low g centrifugation. Beads were blocked in 10 × volume PBS, 200 μg/mL sonicated herring DNA, and 1.5 % BSA at RT and washed in ChIP dilution buffer. Extracts were pre-cleared with 20 μL of blocked anti-HA beads at rotation for 30 min before immunoprecipitation with blocked anti-V5 and anti-HA beads over night at 6 °C. Beads were collected by centrifugation and washed twice in low salt buffer (0.1 % SDS, 1 % Triton-X 100, 2 mM EDTA, 20 mM Tris-HCL, pH 8.1, 150 mM NaCl, and proteinase inhibitors [Roche]), twice in high buffer (0.1 % SDS 1 % Triton-X 100, 2 mM EDTA, 20 mM Tris-HCL pH 8.1, 500 mM NaCl, and proteinase inhibitors [Roche, Mannheim, Germany]), once in LiCl buffer (0.25 M LiCl, 1 % IGEPAL-CA630, 1 % deoxycholixacid sodium salt, 1 mM EDTA, 10 mM Tris-HCl pH 8.1, and proteinase inhibitors [Roche, Mannheim, Germany]), and twice in TE buffer. Elution was performed in 500 μL freshly made elution buffer (1 % SDS, 0.1 M NaHCO_3_, H_2_O). A total of 20 μL of 5 M NaCl was added to eluates and incubated for 4 h at 65 °C for reversing cross-links. A total of 10 μL of 5 M EDTA, 20 μL of 1 M Tris-HCl pH 6.5, and 2 μL of proteinase K (Finnzymes, F-202S, 20 mg/mL) was added to eluates and incubated for 1 h at 45 °C. Phenol/chloroform and isopropanol (v/v) was added, mixed, and centrifuged. A total of 2 μL of glycogen (Sigma Aldrich, cat. no. G1767) and 0.7 × volume isopropanol was added to supernatant and incubated at –20 °C overnight. Precipitated DNA was collected by centrifugation at maximum speed for 30 min at 4 °C. Pellet was carefully washed in 70 % ethanol, air-dried, and dissolved in 50 μL redistilled water. ChIP DNA concentrations were measured on a microplate reader (Thermo Fisher, Flouroskan Ascent FL) by fluorescence of double-stranded DNA (dsDNA) (Lifetechnologies/Invitrogen, Qant-iT™ picogreen, cat. no. P7589). All ChIP DNA was diluted to the same concentration before quantitative real-time PCR.

### ChIP sequencing

DNA libraries were prepared using the ChIP-seq DNA Sample Prep kit (Illumina; IP-102-1001) and sequenced on a Genome Analyzer IIx. The sequence reads were aligned to the human genome (hg19), through the Galaxy project portal [16], using the Illumina Analysis Pipeline. Sequenced reads were mapped with Bowtie [17] allowing one mismatch. Peak calling was performed using the Model-based Analysis of ChIP-Seq v1.4 (MACS) [18], through the Galaxy project portal. Settings: distance = 100; and otherwise standard settings. Ensemble (GRCh37.p13) known genes and transcripts were used as reference gene annotations to identify promoter target genes (PTGs).

### RNA and complementary DNA from cell culture

Cultured cells were lysed using Qiashredder columns (Qiagen, Hilden, Germany) and RNA was purified using the RNAeasy Mini kit (Qiagen) according to the manufacturer’s protocol. RNA concentrations were determined using a NanoDrop 1000 version 3.7.1 (Thermo Fisher Scientific, Waltham, MA, USA). RNA integrity was assessed by 1 % agarose gel electrophoresis. A complementary DNA (cDNA) library was generated from 1 μg of RNA using the iScript™ cDNA Synthesis Kit (Bio-Rad Laboratories, Hercules, CA, USA). All steps were performed according to the manufacturer’s protocol using a mixture of random hexamers and Oligo-dT primers provided with the kit.

### Microarray expression profiling

Microarray (Affymetrix U219) and RNA quality control was performed at Aros Biotechnology, Skejby. Further information is provided elsewhere [19, 20]. A threshold of 1.5 was set for the fold change of microarray probes (Additional file 2).

### Quantitative real-time PCR

Quantitative real-time PCR was performed on the LightCycler®480 (Roche Applied Science, Mannheim, Germany) system. A total of 96 or 384 well plates, with a respective total reaction volume of 10 μL or 5 Μl, were used, respectively. Fluorescence of dsDNA was determined by addition of SYBR®Green (Roche Applied Science, Mannheim, Germany) to the reactions in the concentration described by the manufacturer. The standard settings on the LightCycler software were used for excitation and detection of fluorescence. Primer3 [21] was used in the design of primers (MWG Operon, Ebersberg, Germany).

### Gene set enrichment analysis

We used publicly available summary statistics from single-marker GWASs [4, 22–31] considering only variants outside the broad MHC-region (chr6:25 M-35 M) [28] and filtered for info score ≥0.8 if available.

Data on schizophrenia, bipolar disorder, anorexia nervosa, autism, MDD, ADHD, and cross-disorders were accessed and downloaded via the PGC website [32]. Data on rheumatoid arthritis were downloaded from [33]. Data on coronary artery disease/myocardial infarction have been contributed by CARDIoGRAMplusC4D investigators and have been downloaded from [34]. Data on type 2 diabetes have been contributed by DIAGRAM and downloaded from [35]. Data on Crohn’s disease have been contributed by IIBDG and downloaded from [36]. Data on Alzheimer’s were downloaded from [37] – International Genomics of Alzheimer’s Project (IGAP) is a large two-stage study based upon GWAS on individuals of European ancestry. In stage 1, IGAP used genotyped and imputed data on 7,055,881 single nucleotide polymorphisms (SNPs) to meta-analyze four previously published GWAS datasets consisting of 17,008 Alzheimer's disease cases and 37,154 controls (The European Alzheimer’s Disease Initiative – EADI; the Alzheimer Disease Genetics Consortium – ADGC; The Cohorts for Heart and Aging Research in Genomic Epidemiology Consortium – CHARGE; The Genetic and Environmental Risk in AD Consortium – GERAD). In stage 2, 11,632 SNPs were genotyped and tested for association in an independent set of 8572 Alzheimer’s disease cases and 11,312 controls. Finally, a meta-analysis was performed combining results from stages 1 and 2. For the risk gene enrichment analysis, we used MAGMA [38] and default settings. Genes were annotated using Ensemble (GRCh37.p13, same reference for identifying BRD1 PTGs). Information about the genetic correlation pattern in the data (linkage disequilibrium) was obtained using the 1000 Genomes European panel [39]. To assess whether or not the BRD1 protein network was enriched with de novo mutations with relevance to autism risk [40, 41] or rare mutations with relevance to schizophrenia risk [42], we compared the proportion of mutations in the genes among cases to controls (obtained from the referenced studies) using a one-sided binominal test correcting for the overall ratio of mutations in cases compared to controls.

### Identification of spatiotemporal networks in the human brain

Human brain transcriptome (RNA-seq) data were obtained from www.brainspan.org [43]. We filtered the dataset including only genes with coefficient of variance more than 0.1 thereby removing genes with little or no information with regards to the spatiotemporal dynamics. Since the BRD1 network contained both protein-coding and non-protein-coding genes, both types of genes were included in the analysis yielding 21,396 uniquely identified genes. Data were segregated into 32 spatiotemporal intervals consisting of eight temporal intervals (P1–P8) and four brain regions (R1–R4). Brain regions (R) were grouped according to transcriptional similarities based on the hierarchical clustering described here [44]. Temporal intervals (P) were grouped as follows: P1 included post-conceptual week (pcw) 8–13 (first trimester); P2 included 16–26 pcw (second trimester); P3 included 35–37 pcw (third trimester), P4 included 4–10 months; P5 included 1–4 years; P6 included 8–13 years; P7 included 15–19 years; P8 included 21–40 years. The fraction of co-expressed genes (correlation coefficient >0.5) was calculated for each spatiotemporal interval and each BRD1 sub-network and compared to the background of genes co-expressed with BRD1 in the entire dataset. To determine whether or not the fraction of co-expressed genes was significantly higher than the background, a one-sided binominal test was performed while adjusting the *P* value for the number of tests performed (Bonferroni correction).

### Software and statistical analysis

Ingenuity Pathway analysis was used for the bioinformatics analysis. Simulations, statistical analysis, and graphical illustrations were conducted in R, Python, Excel (Microsoft, Office 2010, USA), and GraphPad Prism 5.

### Availability of data

The ChIPseq and microarray data discussed in this publication have been deposited in NCBI's Gene Expression Omnibus [45] and are accessible through GEO Series accession numbers GSE62811 (https://www.ncbi.nlm.nih.gov/geo/query/acc.cgi?acc=GSE62811) and GSE79255 (https://www.ncbi.nlm.nih.gov/geo/query/acc.cgi?acc=GSE79255). The protein-protein interactions from this publication have been submitted to the IMEx consortium [46, 47] through IntAct [48] and assigned the identifier IM-24990.

## Results

### Identification of BRD1 protein-protein interactions

**Fig. 1:**
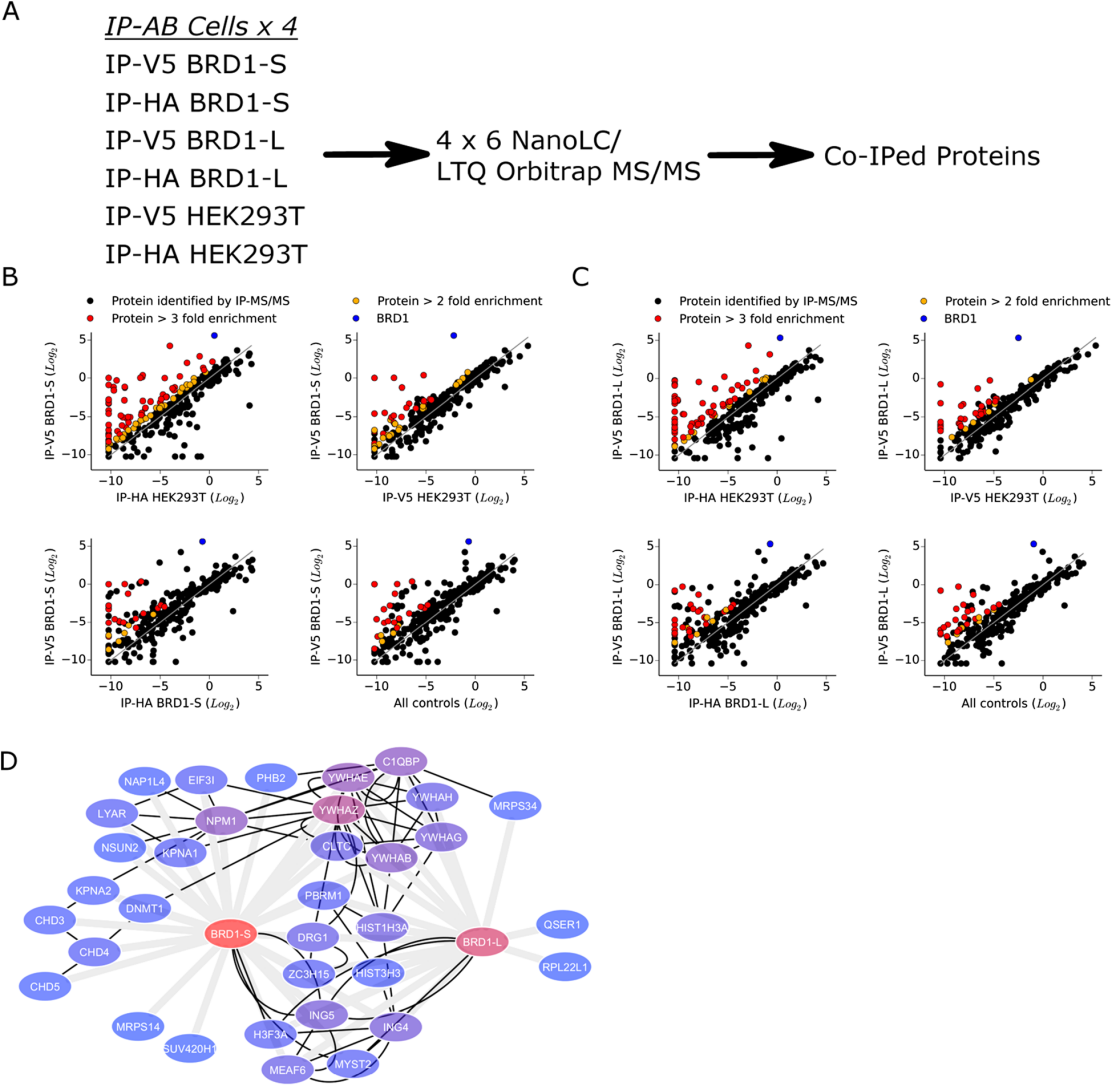
Co-immunoprecipitation (co-IP) coupled to mass spectrometry MS/MS analysis of BRD1-S and BRD1-L. **a** co-IPs were performed on cell extracts harvested from BRD1-S, BRD1-L, and HEK293T cell lines, using either anti-V5 antibody conjugated (IP-V5) or anti-HA antibody conjugated (IP-HA) beads. Cell culturing, extract preparation, and co-IP were repeated four times before preparation for mass spectrometry analysis (for further details, see “Methods”). **b**, **c** The combined spectral intensity of each individual protein was normalized to the mean intensity of all the proteins for each sample or control. In total, 319 proteins were identified from at least one sample or control in the analysis. The *plots* represent the normalized (Log_2_) spectral intensities of each identified protein (*black*) plotted as sample (*y-axis*) against control (*x-axis*). BRD1 (*blue*) was identified as the most enriched protein in the BRD1-S (**b**) and BRD1-L (**c**) pull-downs. The *plots*, from the *upper right* to the *lower left*, illustrate a stepwise sorting of proteins that showed > two- or > threefold enrichment (*red* and *orange*, respectively, and otherwise *black*) in the sample compared to controls. The last *plots* in (**b**) and (**c**) show the proteins that were identified as enriched in all controls, where the *x-axis* represents the mean of all the control experiments. These proteins were then further divided into three levels of co-IP strength taking into account the reproducibility of the co-IP within the repeated experiments (see Table 1). **d** BRD1 PPI network. *Gray lines* represent the interactions identified in this study by co-IP LC-MS/MS and *black lines* represent experimentally verified PPIs annotated in publicly available databases (BioGRID, CCSB Human Interaction Network Database, DIP, HPRD, IntAct, MINT, BIND, MPPI, and GNP). A high level of *red* in nodes indicates a high number of edges while a high level of *blue* indicates a low number of edges

In order to identify PPIs of the two BRD1 isoforms (BRD1-S and BRD1-L), we generated HEK293T cells stably expressing either V5 epitope tagged BRD1-S or BRD1-L (referred to as BRD1-S and BRD1-L cells). Both epitope tagged BRD1 isoforms localized to the nucleus of the HEK293T cells (Additional file 1), suggesting that the V5 epitope does not disrupt nuclear localization of the proteins. By co-immunoprecipitations, followed by identification of associated proteins using nanoLC coupled to a LTQ Orbitrap MS/MS system (Fig. 1a–c), we identified 24 and 16 PPIs for BRD1-S and BRD1-L, respectively (Table 1). The previously reported BRD1 interaction partners MEAF6, ING4, and ING5 [7, 49] were identified to interact with both BRD1-S and BRD1-L, whereas MYST2 [7] was found to interact solely with BRD1-S (Fig. 1d and Table 1). Likewise, our finding that histone H3 interacts with BRD1-L (Table 1) is in line with a previous study that identified the unmodified N-tail of histone H3 to interact with the PHD1 domain of BRD1 [50]. We cross-referenced the identified protein networks with public databases (BioGRID, CCSB Human Interaction Network Database, DIP, HPRD, IntAct, MINT, BIND, MPPI, and GNP) and discovered 20 novel BRD1-S PPIs and 13 novel BRD1-L PPIs which have not previously been reported, some of which were identified solely for one isoform (Fig. 1d and Table 1). A recent large-scale protein-protein interaction study found that the majority of protein isoforms share less than 50 % of their interaction partners [51]. In line with these results, 62.5 % of the identified PPIs were found to be common among the two BRD1 isoforms. As an example, the Histone-lysine N-methyltransferase protein suppressor of variegation 4-20 homologue 1 (SUV420H1) was identified as a specific PPI for BRD1-S (Fig. 1d and Table 1). As part of the list of common BRD1 PPIs (i.e. interacting with both BRD1-S and BRD1-L), we identified the 14-3-3 tyrosine monooxygenase proteins YWHAE, YWHAH, YWHAZ, and the poly bromo 1, PBRM1 (Table 1). These PPIs are of particular interest since genetic association studies have previously implicated *YWHAE*, *YWHAH*, and *YWHAZ* with schizophrenia and bipolar disorder [52–55] and the *PBRM1* locus has surpassed genome-wide significance in both schizophrenia and bipolar disorder GWASs [4, 56–58]. We confirmed the interaction between BRD1 (for both isoforms) and PBRM1, by co-IP with antibodies against endogenous PBRM1 followed by western blot detection of BRD1 (Additional file 3).

### Identification of chromatin-binding sites of BRD1 by ChIP sequencing

**Fig. 2:**
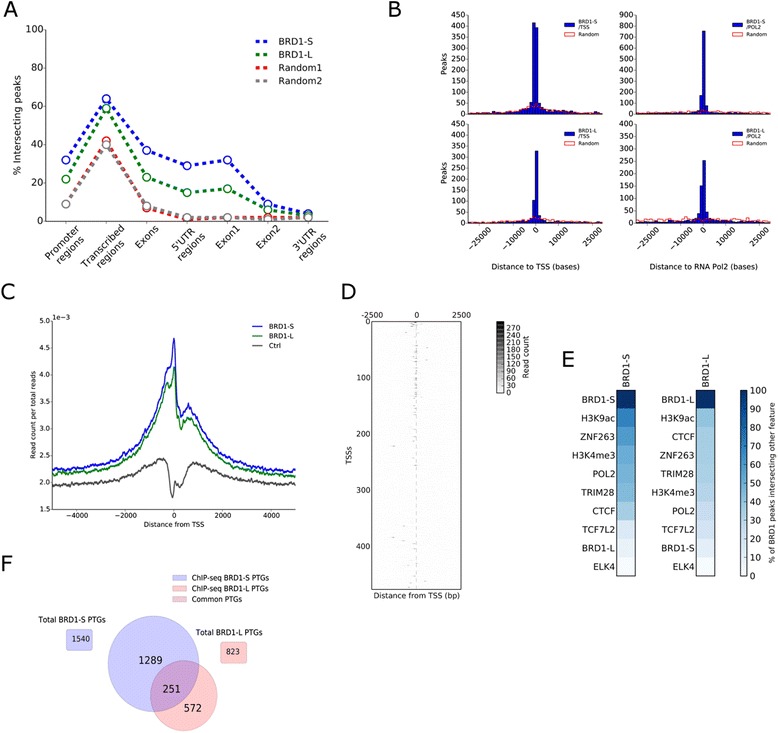
ChIP sequencing analysis of BRD1-S and BRD1-L. **a** The percentages of BRD1-S and BRD1-L ChIPseq peaks that intersect promoter regions of protein-coding genes (defined as 5 kb upstream from transcription start site), transcribed regions (all exons, introns, and UTRs), exons (all exons), 5’UTR, exon 1 exon 2, and 3’UTR, were identified and shown by the *perforated blue and green lines* in the graph. Random peak regions, comprising the same chromosome distribution and region sizes as the BRD1 ChIPseq regions, were generated and shown as Random 1 (2205 regions, *red*) and Random 2 (1722 regions, *gray*). **b** The minimum relative distance from a BRD1-S and BRD1-L ChIP-seq region to transcription start sites (*left panel*) or to RNA Pol II binding sites (*right panel*) was identified for all 2205 (BRD1-S) and 1722 (BRD1-L) regions and illustrated as the *blue histograms*. Random peak regions comprising the same chromosome distribution and region sizes as the BRD1 ChIPseq regions were generated and shown as *red line histograms* (Random). **c** In order to generate a profile of BRD1-S (*blue*), BRD1-L (*green*) binding to the TSS of genes, we identified and plotted the number of sequenced reads per total reads across a window of +/– 5 kb from the TSS of all protein-coding genes in the human genome. The control (Ctrl, *black*) represents ChIP-seq using an antibody against the HA-epitope. **d** A *heatmap* of read counts from BRD1-S ChIP-seq across a +/– 2.5 kb window from the TSS of genes located on chromosome 1 and 10. Only regions with high read counts are shown here. **e** ChIPseq regions of chromatin-binding proteins and histone marks, identified in HEK293 cell lines, were obtained from the ENCODE database and from a histone H3K9ac dataset [59]. The percentages of BRD1-S and BRD1-L ChIPseq peak regions that intersected with the binding of other chromatin-binding proteins or the location of histone marks were identified and illustrated as *heatmaps*. **f** Genes comprising transcription start sites in a window of +/– 5 kb from a BRD1-S or BRD1-L ChIP-seq peak region were identified as promoter target genes (PTGs). *Venn diagram* illustrates the number of PTGs identified for BRD1-S and BRD1-L

To identify BRD1 chromatin interactions and target genes, we conducted chromatin immunoprecipitation of epitope tagged BRD1-S and BRD1-L from the cell lines described above (Additional file 4) followed by next generation DNA sequencing (ChIP-seq) generating on average 24 million mapped reads (Additional file 5). We identified 2205 and 1722 ChIP-seq peaks for BRD1-S and BRD1-L, respectively. The identified peaks intersected more with promoter regions (defined as the region from a transcription start site and 5 kb upstream), transcribed regions (all exons, introns, and UTRs), exons (all exons), 5’UTR, and exon 1 of protein-coding genes compared to random genomic regions of the same sizes and chromosome distributions (Fig. 2a). This was not observed for 3’UTR regions and exon 2, providing evidence that the increased intersection with transcribed regions, is caused by frequent intersections with 5’UTR and/or exon 1 regions (Fig. 2a). Furthermore, BRD1-S and BRD1-L peaks were predominantly located near transcription start sites (TSSs) of genes (Fig. 2b, left panel). In order to gain more insights as to where BRD1-S and BRD1-L primarily bind at TSSs, we counted the number of sequencing reads obtained from the ChIP-seq data across a window of +/– 5 kb from TSS for all protein-coding genes in the human genome. This approach showed that the majority of sequencing reads aligned upstream (0 to –2 kb) from TSS of genes while another, smaller part of the reads aligned downstream (0 to 2 kb) from TSS of genes (Fig. 2c) suggesting that BRD1-S and BRD1-L bind mainly upstream but also downstream of TSS of genes. To further investigate whether BRD1 binds in a co-occurring manner at TSSs, we generated a heatmap of BRD1-S ChIP-seq read counts in TSSs from chromosome 1 and 10 (Fig. 2d). The analysis did not indicate that BRD1 binding was occurring twice at the same TSS. To evaluate the functional role of BRD1-S and BRD1-L, we compared all ChIP-seq regions to ChIP-seq regions of histone marks [59] and other chromatin binding proteins [60] and generated genomic binding profiles for both isoforms (Fig. 2e and Additional file 6). Both BRD1-S and BRD1-L were found to bind regions that highly overlap with histone H3K9ac genomic regions. Interestingly, the histone H3K9ac level was previously found to be reduced in erythroblasts (CD71^+^ Ter119^–^) isolated from *Brd1*^*–/–*^ mouse embryos [7]. Furthermore, large subsets of BRD1-S and BRD1-L ChIP-seq regions were found in close proximity of or directly overlapping with RNA polymerase II binding sites (Fig. 2b, right panel and E). In view of the aforementioned results, predominant binding at RNA polymerase II binding sites, histone H3K9ac sites, promoter regions, 5’UTR, exon 1, and TSSs suggest that BRD1 primarily binds to actively transcribed regions of the genome. We observed a substantial proportion of the BRD1-S and BRD1-L peaks to be located at bi-directional/head-to-head genes that may share promoter or enhancer elements. Binding of BRD1 at these sites potentially affects transcription of both genes. Consequently, we defined BRD1 PTGs as genes having TSS within a window of +/– 5 kb from a BRD1 ChIP-seq peak. Overall, we identified sets of 1540 and 823 PTGs for BRD1-S and BRD1-L, respectively, with 251 PTGs being common to both isoforms (Fig. 2f). Among these, we validated five by ChIP followed by quantitative real-time PCR (Additional file 7 and Additional file 8).

### Pathway analysis of the BRD1 interaction network

To further characterize the identified BRD1 interaction network, we used the Ingenuity Pathway Analysis (IPA) software to identify enrichment of specific pathways. Three datasets (BRD1-S PTGs + PPIs, BRD1-L PTGs + PPIs, and the combined BRD1 PTGs + PPIs) were analyzed according to gene categories (diseases and disorders, molecular and cellular functions, and physiological system development functions) and pathways as defined by IPA. After correction for multiple testing, we identified two, one, and 21 significant IPA categories, as well as, 14, six, and 27 significant canonical pathways among the three tested BRD1 interaction networks, respectively (categories, pathways, and genes within these can be found in Additional file 9). The most significant category in all three BRD1 interaction networks was the gene-expression category. Of neuropsychiatric relevance, we identified the abnormal morphology of neural arch and malformation of brain as significant sub-categories when analyzing the full set of BRD1 PTGs + PPIs. The most significant pathways identified for the subsets of BRD1-S and BRD1-L were IGF-1 Signaling and Cell Cycle: G1/S Checkpoint Regulation, respectively. Together the results indicate that BRD1 is a regulator of transcriptional processes and that it is involved in pathways important for brain development.

### Identifying differentially expressed genes following changes in BRD1 expression levels

To identify differentially expressed genes following expression level changes in BRD1, we extracted RNA from BRD1-S and BRD1-L cells and parental HEK293T cells (Additional file 10) as well as from HEK293T cells transfected with siRNA directed against *BRD1* or scrambled siRNA (Additional file 11). Microarray expression analyses covering 17,051 genes were performed on cDNA libraries constructed from the RNA samples (Additional file 2). Combining the results from both BRD1 up- and downregulation studies yielded a list of 4643 differentially expressed genes (DEGs), defined as having a probe fold change of more than 1.5, and corresponding to 27 % of the genes covered by the microarray. We subsequently examined whether the identified DEGs were enriched within BRD1 PTGs. For both BRD1-S and BRD1-L, DEGs accounted for 33 % of all PTGs, which represents a significant enrichment (*P* <0.001; Additional file 12). The observed enrichment supports that BRD1 binding at TSSs takes part in regulating gene expression.

### Exploring the gene-regulatory potential of BRD1-S and BRD1-L isoforms

**Fig. 3:**
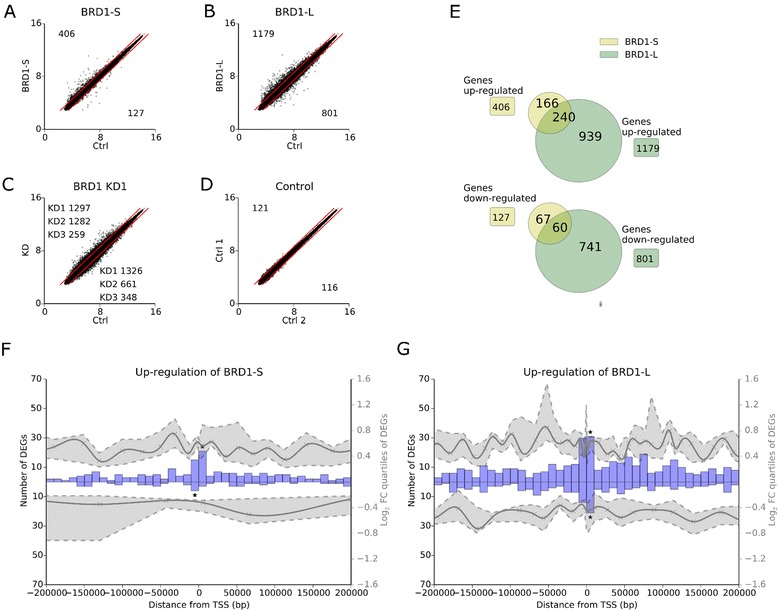
Integration of ChIP-seq data and gene-expression data. **a** Examples of gene expression arrays performed. Shows (**a**) upregulation of BRD1-S vs. control, (**b**) upregulation of BRD1-L vs. control, (**c**) siRNA downregulation of BRD1 vs. control, and (**d**) two controls transfected with scrambled siRNA, control 1 vs. control 2. The *red lines* indicate fold change 1.5 which was used in this study to identify DEGs. The number of upregulated and downregulated DEGs is shown for each study in the *upper left and lower right corner*, respectively. Three siRNA knockdown experiments of BRD1 (KD1-3) were performed. More information about KD1-3 can be found in Additional file 10. **e** The number of genes that overlap after upregulating BRD1-S and BRD1-L expression. After upregulating either BRD1-S or BRD1-L, the number of DEGs was identified in 10 kb segments (*blue bars*) across a window of +/– 200 kb from either (**f**) BRD1-S or (**g**) BRD1-L binding sites, respectively. The expression fold change quartiles were obtained from segments of 10 DEGs. *Gray areas* indicate the upper and lower quartiles while the middle is shown as a *gray line*

In order to explore the gene-regulatory potential of BRD1-S and BRD1-L isoforms, we performed integrative analyses of ChIP-seq and gene expression data. The number of up- and downregulated DEGs according to expression level from each study (BRD1-S or BRD1-L upregulation or BRD1 siRNA-mediated downregulation) is illustrated in Fig. 3a–d. Also, we investigated the overlap of DEGs identified after upregulating either BRD1-S or BRD1-L (Fig. 3e). We then created a window including +/– 200 kb from each BRD1 binding site, fragmented the window in 10 kb segments, and determined the number of DEGs having TSSs in these segments. The analysis was performed for DEGs identified after upregulating BRD1-S or BRD1-L or downregulating endogenous BRD1 (Fig. 3f, g, Additional file 13). Significantly more up- or downregulated genes were identified in segments where BRD1-S or BRD1-L binds in close proximity to TSSs compared to all segments across the window (*P* <0.001) supporting that BRD1-S and BRD1-L are involved in regulating expression of genes when bound in close proximity to the TSSs. Within these segments, upregulation of either BRD1-S or BRD1-L resulted in more upregulated compared to downregulated genes while downregulating BRD1 resulted in more downregulated compared to upregulated genes. Furthermore, upregulation of BRD1-L proportionally generated more downregulated genes compared to BRD1-S in these segments (Fig. 3f, g). We conclude that both BRD1-S and BRD1-L primarily play a role in activation of gene expression when bound near TSSs, although they may act, and BRD1-L in particular, as a repressor for a subset of genes. Interestingly, for BRD1-L upregulated DEGs we observed the additional feature that increased expression fold changes were present when having the TSS either –50 kb upstream or 100 kb downstream the BRD1-L binding site (Fig. 3g). This suggests that BRD1-L may also participate in activating gene expression distal from its binding sites.

In order to investigate BRD1 gene-regulatory potential while considering all genes on the array (and not only DEGs), we segmented and calculated the average fold change of all probes within windows +/– 10 kb and +/– 200 kb from BRD1 binding sites similarly as in the previous analysis. Overexpressing BRD1-S primarily increased the overall expression fold change of genes with BRD1-S binding approximately +/– 1 kb from their TSSs while downregulating BRD1 reversibly downregulated genes within the same range (Additional file 13). Overexpressing BRD1-L resulted in increased expression of genes having TSSs –50 kb upstream and +100 kb downstream of BRD1-L binding similar to what we observed in the previous analysis of the DEGs; however, a clear reverse pattern was not observed after downregulating BRD1 (Additional file 13).

The integrative analysis of ChIP-seq and gene expression data collectively suggests that BRD1 can activate and more rarely, repress, gene expression by binding to TSSs, as well as suggesting that specifically the BRD1-L isoform plays a role in activating gene-expression distal from its chromatin binding sites.

### Pathway analysis of DEGs identified as a consequence of upregulating BRD1-S and BRD1-L

To elucidate potential functional consequences of the BRD1-mediated changes in gene expression and identify potential differences between BRD1-S and BRD1-L isoforms, the DEGs identified by upregulating either BRD1-S or BRD1-L were analyzed by IPA software to identify enrichment of specific canonical pathways. DEGs were defined as previously stated using a 1.5 threshold on the probe fold change. The top ten most significant canonical pathways can be found in Additional file 14. The genes in all the pathways were mainly upregulated. The most significant canonical pathway identified when upregulating BRD1-S or BRD1-L was Reelin Signaling in Neurons and IL-8 Signaling, respectively. Of notice, the IGF1 Signaling pathway which was identified to be the most significant pathway among the BRD1-S interaction network was also identified among the ten most significant pathways of the BRD1-S DEGs further implicating BRD1-S with IGF1 signaling. Among the identified pathways, the Reelin Signaling in Neurons pathway is of a particular interest to brain function and development [61].

### Correlation of the BRD1 interaction network with spatiotemporal *BRD1* expression profiles in the human brain

**Fig. 4:**
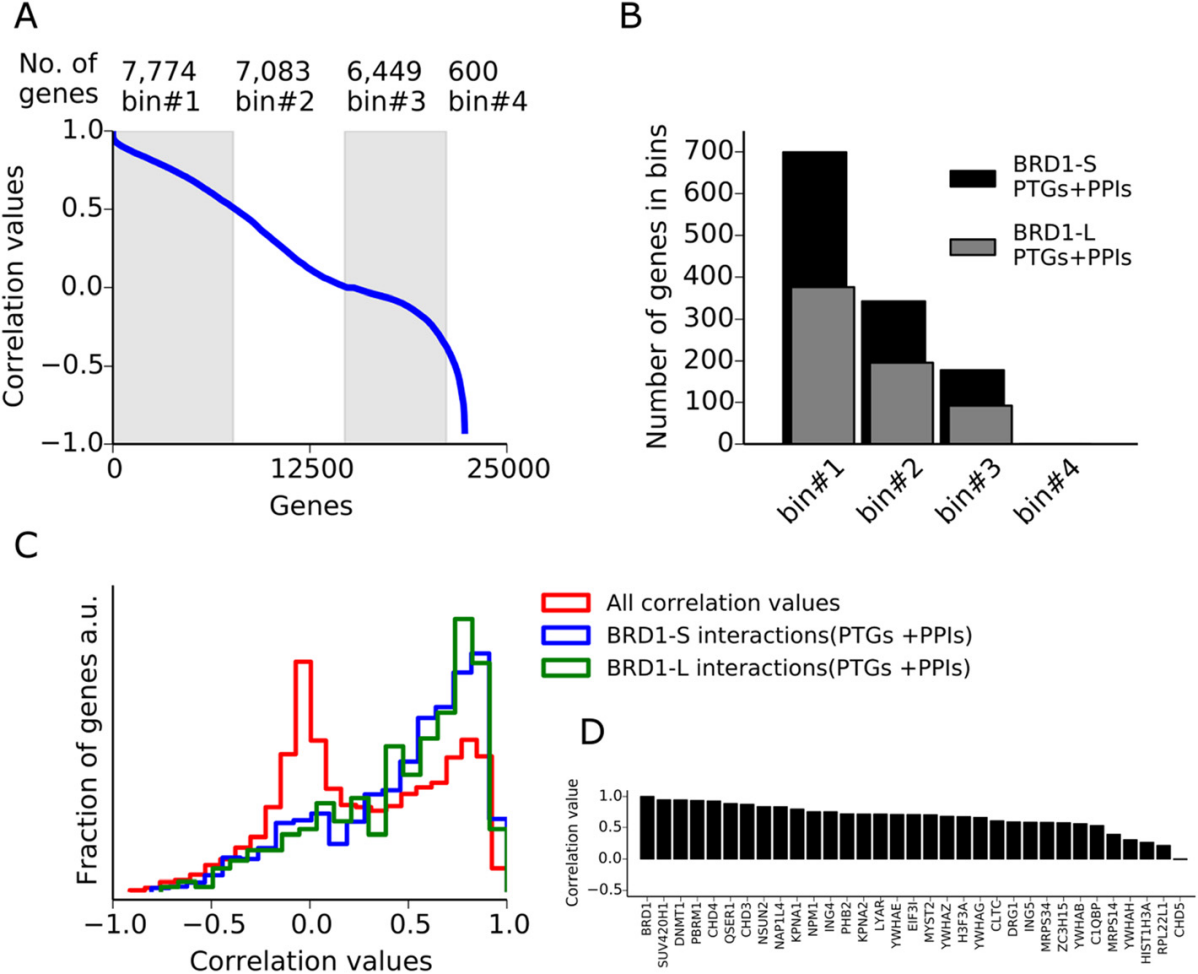
Co-expression analysis of the BRD1 interaction network in human brain. **a** A total of 21,906 gene-correlation values ranging from 1 to –1 were obtained across 13 developmental stages and 26 different brain regions from the Brainspan RNA-seq database. Correlation values for all genes were illustrated as a *blue line* and *gray and white bins* (#1–4) define sections (1 to 0.5, 0.5 to 0, 0 to –0.5, and –0.5 to -1) where the number of genes in each bin was shown at the top. **b** From the identified BRD1-S and BRD1-L PTGs + PPIs, 1241 and 672 were identified in the database, respectively. The correlation values for each gene distribute into bins #1–4 as illustrated. **c** The distributions of the correlation values of all genes in the Brainspan database and the correlation values of BRD1-S and BRD1-L PTGs + PPIs (BRD1-S and BRD1-L interaction networks) were summarized as relative to the number of genes and illustrated in a histogram (a.u. = arbitrary units). Mann–Whitney tests were performed for correlation values from either of the BRD1-S or BRD1-L interaction networks and all genes identified. Both tests resulted in the rejection of the null hypothesis of identical medians (*P* < 1 × 10^–10^). **d** Correlation values for the BRD1 PPIs represent the level of co-expression with *BRD1* in the developing human brain

In order to explore the role of BRD1 in the human brain, we obtained temporal RNA-seq profiles and expression array profiles from the Brainspan atlas of the developing human brain [43] and the Human Brain Transcriptome database [62], respectively. A high *BRD1* expression was observed in the fetal stages (until approximately day 180) for all brain regions whereas a high *BRD1* expression in the cerebellar cortex was observed throughout the timeline of the datasets (age >40 years; Additional file 15). To investigate whether the identified BRD1 interaction network is likely to operate in the context of the human brain, we used data from the Brainspan RNA-seq database to correlate the expression of 21,906 genes with *BRD1* expression across 13 developmental stages and 26 different brain regions in the human brain. Correlation values (Fig. 4a) for all genes ranged from 1 (complete correlation with *BRD1* expression) to –1 (complete anti-correlation with *BRD1* expression). In total, 1241 and 672 interaction partners (PTGs + PPIs) for BRD1-S and BRD1-L, respectively, were identified in the Brainspan database (Fig. 4b). We observed significantly more correlation for both of the BRD1 isoforms than expected by chance (Fig. 4c), indicating that the BRD1 interactions are likely to occur also in the human brain. The PPIs identified for BRD1-S and BRD1-L were also more correlated with *BRD1* expression (*P* <1 × 10^–10^ and *P* = 0.0039, respectively); in particular, *SUV420H1*, *DNMT1*, *PBRM1*, and *CHD4* were highly correlated with *BRD1* (Fig. 4d).

### Disease risk enrichment analysis of the BRD1 interaction network

**Fig. 5:**
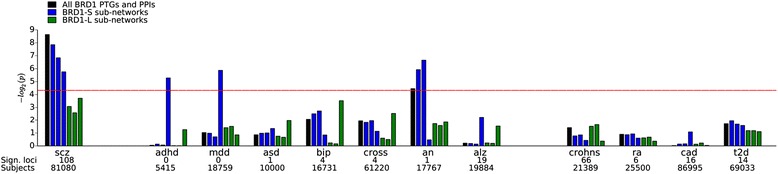
Disease risk gene analysis of the BRD1 network. Enrichment for genetic disease risk was investigated for seven BRD1 sub-networks across 12 GWASs comprising eight brain disorders and four disorders that are not considered brain disorders. The *bars* indicate BRD1 sub-networks in the order: all BRD1-S and BRD1-L PTGs and PPIs (*black*), BRD1-S PTGs and PPIs, BRD1-S PTGs, BRD1-S PPIs, BRD1-L PTGs and PPIs, BRD1-L PTGs, and BRD1-L PPIs. GWAS from the left are: schizophrenia (*scz*) [4, 32], attention deficit hyperactivity disorder (*adhd*) [27, 32], major depressive disorder (*mdd*) [28, 32], autism spectrum disorder (*asd*) [32], bipolar disorder (*bip*) [30, 32], psychiatric cross-disorders (*cross*) [25, 32], anorexia nervosa (*an*) [23, 32], Alzheimer’s disease (*alz*) [24, 37], Crohn’s disease (*crohns*) [36], rheumatoid arthritis (*ra*) [29, 33], coronary artery disease (*cad*) [31, 34], and type 2 diabetes (*t2d*) [26, 35]. The *red line* indicates *P* = 0.05, before correction for the number of GWAS tested. The number of significant loci as well as the number of subjects in each study is noted under each study

Using available GWAS datasets obtained from the Psychiatric Genomics Consortium (PGC) we investigated whether the BRD1 interaction network is enriched for mental disorder risk applying the MAGMA program [38]. As the evidence for genetic association to the *BRD1* locus is particularly strong in schizophrenia, we tested for enrichment in this disorder as our main analysis. Out of the entire BRD1 network, 1853 genes remained after filtering SNPs and removing genes in the MHC region (see Methods), 468 of these had gene-wide *P* values <0.05, and 44 of these were significant after adjusting for the number of genes tested (Additional file 16). In this analysis the entire BRD1 network showed significant enrichment (Fig. 5).

In order to explore whether the interaction network might also be enriched for susceptibility to other mental disorders, we tested 12 other GWAS datasets, comprising eight psychiatric disorders and four disorders not considered related to brain function. In addition, to investigate whether the risk enrichment is dependent on the specific isoform, we stratified for BRD1-S and BRD1-L sub-networks. When adjusting for the number of GWASs tested, significant enrichment was observed solely for the entire BRD1 network in schizophrenia (*P*_*adjusted*_ = 0.03, Fig. 5). Interestingly, substantial differences in enrichment patterns between the two BRD1 isoforms were observed in several disorders. The BRD1-S sub-networks comprising PTGs showed enrichment for schizophrenia and anorexia nervosa while the PPI sub-network showed evidence of enrichment for attention deficit hyperactivity disorder (ADHD) and major depressive disorder (MDD) risk before adjusting for the number of tests (*P* <0.05). These results suggest that the BRD1-S network may play a larger role in mental disorder risk compared to the BRD1-L network.

Finally, we explored whether the network was enriched for de novo mutations and rare disruptive variants observed in autism and schizophrenia [40–42]. The analysis did not identify significant enrichment. However, in particular among the PPI network genes, we did observe de novo mutations in autism probands that were not identified in healthy siblings, including one missense mutation in *BRD1*, one missense mutation in *CHD4*, and four disruptive and two missense mutations in *SUV420H1* [40, 41], as well as rare disruptive mutations seen only in schizophrenia cases and not in controls (in *BRD1*, *ING5*, *YWHAZ*, *NAP1L4*, *MYST2*, and *HIST1H3A*) [42].

In summary, we identify the BRD1 interaction network to be enriched for schizophrenia risk thereby providing supporting evidence that BRD1 plays a role in the etiology of schizophrenia. Additionally, our analyses suggest that the BRD1-S interaction network is more enriched for mental disorder risk, including schizophrenia risk, compared to the interaction network of BRD1-L.

### Exploring the BRD1 spatiotemporal interaction networks in the human brain

**Fig. 6:**
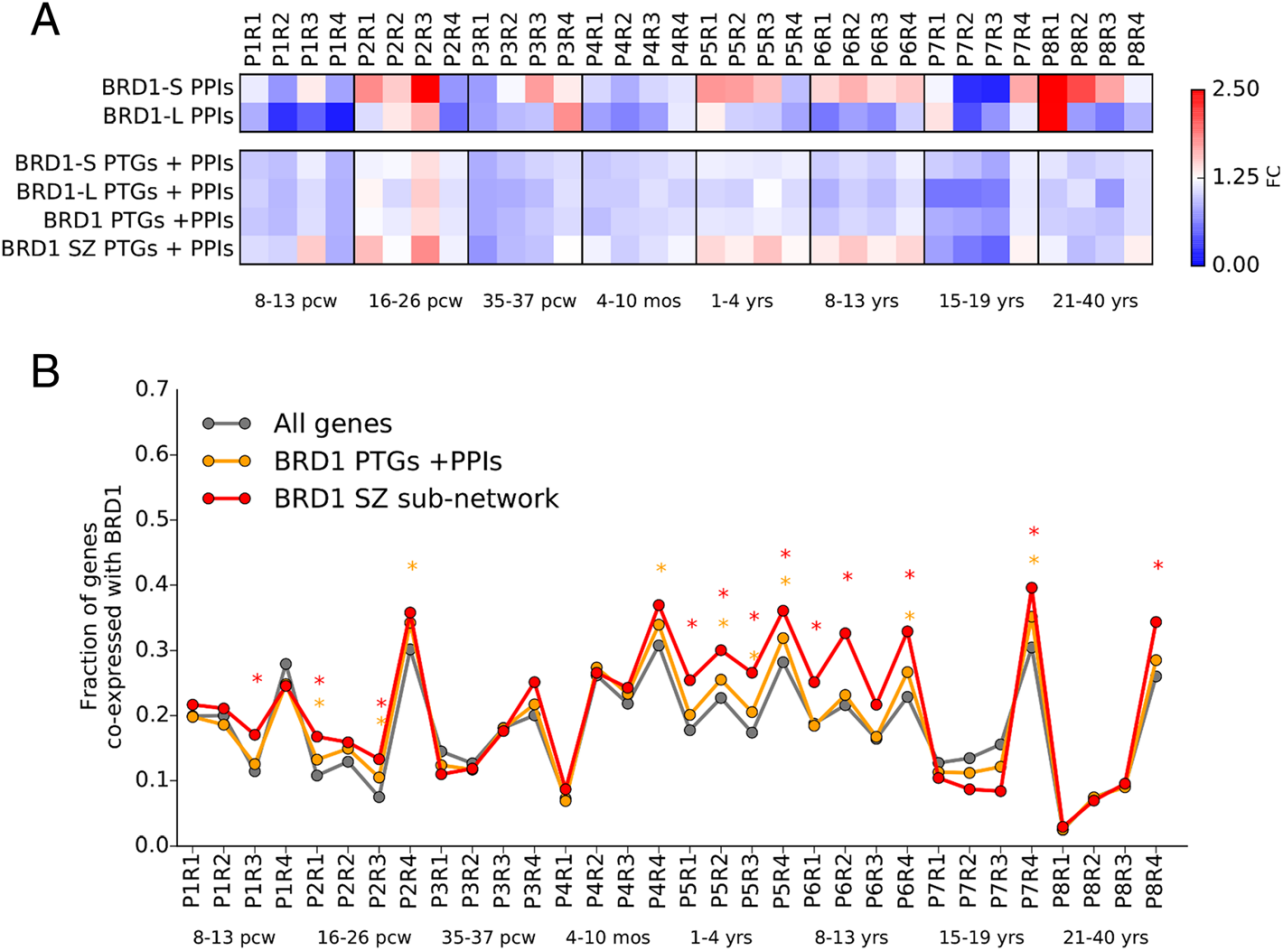
Identifying spatiotemporal networks of BRD1 in the human brain. **a** The fraction of co-expressed genes (correlation coefficient >0.5) was calculated for each spatiotemporal interval and each BRD1 sub-network and compared to the background of genes co-expressed with BRD1 in the entire dataset. The color-coding denotes the fold change of the fraction of co-expressed genes compared to the background. **b** The fraction of genes that co-expressed with BRD1 across each of the 32 spatiotemporal intervals was calculated for all genes (gray lines), genes in the BRD1 PTGs + PPI network (orange lines), and genes in BRD1 schizophrenia network of 468 genes (red lines). A one-sided binominal test was performed for each BRD1 sub-network and spatiotemporal interval compared to the expected fraction in the background where the asterisk (*) denote *P* < 0.05 after adjusting for the number of tests. Temporal intervals (P) were grouped as follows: P1 included pcw 8–13 (first trimester), P2 included 16–26 pcw (second trimester), P3 included 35–37 pcw (third trimester), P4 included 4–10 months, P5 included 1–4 years, P6 included 8–13 years, P7 included 15–19 years, and P8 included 21–40 years. Brain regions (R) were grouped as follows: R1 included the posterior inferior parietal cortex, primary auditory cortex, primary visual cortex, superior temporal cortex, and inferior temporal cortex; R2 included the primary somatosensory cortex, primary motor cortex, orbital prefrontal cortex, dorsolateral prefrontal cortex, medial prefrontal cortex, and ventrolateral prefrontal cortex; R3 included the striatum, hippocampus, and amygdala; and R4 included the mediodorsal nucleus of the thalamus, and cerebella cortex

Taking into consideration that the BRD1 network is enriched with schizophrenia risk and that this enrichment seems to be more pronounced in the BRD1-S network, we asked whether these networks are more likely to be interacting in specific regions and developmental intervals in the human brain. In order to evaluate co-expression with *BRD1* in the human brain, we applied an approach similar to the one described here [63]. We used RNA-seq data from Brainspan to build 32 spatiotemporal intervals comprising eight temporal intervals (P1–P8) and four brain regions (R1–R4) (Fig. 6). We then calculated the fraction of genes that were expressed with Spearman’s correlation coefficient >0.5 compared to *BRD1* for each of the 32 intervals. Previously we showed that the majority of genes in the BRD1 network are positively correlated with *BRD1* expression across all samples in Brainspan, including the BRD1 PPI networks. To gain insight into which spatiotemporal intervals that may be driving this positive correlation, we investigated the fraction of *BRD1* co-expressed genes in the BRD1-S and BRD1-L PPI networks and compared them to the background fraction across all genes in the Brainspan dataset. A higher fraction of co-expressed genes was observed for both networks during pcw 16–26 (P2, second trimester) in the prefrontal cortex regions (R2) and the hippocampus, striatum, and amygdala regions (R3) (Fig. 6a, top panel and Additional file 17). The BRD1-S PPI network was found to have a higher fraction of co-expressed genes across several spatiotemporal intervals including P3R3, P5R1-R3, P6R1-R4, P7R4, and P8R1-3.

We then expanded the analysis to include the entire BRD1 interaction network (BRD1-S and BRD1-L PTGs and PPIs) and the BRD1 schizophrenia sub-network consisting of the 468 genes that achieved a gene-wide *P* value <0.05. The highest fraction of genes co-expressed with *BRD1* consistent across all BRD1 networks was observed for pwc 16–26 (P2) in the hippocampus, striatum, and amygdala regions (R3) (Fig. 6a). All BRD1 PTG + PPI networks showed a significantly higher fraction of genes co-expressed with *BRD1* in this interval (Fig. 6b and Additional file 17). We noticed that the BRD1 schizophrenia sub-network, across several spatiotemporal intervals, was more enriched with genes that co-expressed with *BRD1* compared to other networks that included PTGs (Fig. 6a, bottom panel). These intervals were largely co-occurring with those of the many times smaller BRD1-S PPI network. The BRD1 schizophrenia sub-network comprised a significantly higher fraction of genes that co-expressed with *BRD1* in 12 of the total 32 spatiotemporal intervals (Fig. 6b).

## Discussion

In this study we have obtained results providing novel insights into the molecular interactions of BRD1 and its relation to mental disorders.

### The molecular function of BRD1

Our results provide confirming evidence that BRD1 is part of a histone acetyltransferase complex comprising ING4, ING5, MEAF6, and MYST2 [7, 49] along with supporting evidence for BRD1 and histone H3 interaction [50].

Interestingly, we identify several novel protein interactions that are important for chromatin modulation and gene regulation, including PBRM1 which is part of the SWI/SNF chromatin remodeling complex PBAF [64] and the 14-3-3 proteins YWHAE, G, H, and Z. Among several known functions, 14-3-3 proteins facilitate the recruitment of transcription factors to chromatin particularly in conjunction with phosphorylated histone H3S10 and H3S28 [51, 65, 66], e.g. the PBAF complex is recruited to chromatin via the 14-3-3 proteins and phosphorylated histone H3S10 [64]. Our results identify both BRD1 isoforms to bind 14-3-3 proteins as well as PBRM1 indicating that BRD1 plays a role in chromatin remodeling.

Although the identified PPI networks of BRD1-S and BRD1-L seem to comprise a common set of core proteins, we identify several isoform-specific PPIs that could indicate a significant functional difference between the two isoforms. For instance, the histone methyltransferase SUV420H1 was identified only for the BRD1-S isoform and QSER1, a relatively uncharacterized protein, was only identified for BRD1-L. In addition, BRD1-S and BRD1-L had a relatively limited overlap of PTGs as well as DEGs suggesting that these two isoforms, although very similar in amino acid sequence, target and regulate different gene sets. By integrating ChIP-seq and expression data, we were able to show that BRD1-S primarily regulate gene expression in proximity to TSSs while BRD1-L, in addition to regulating gene expression in proximity to TSSs, has the capacity to regulate gene expression further up- and downstream from its binding sites. Based on our observations, it is tempting to speculate that BRD1-L is involved in mediating chromatin loop status thereby regulating the expression of distal genes similar to the mechanisms identified for SATB1 [67]. Conversely, BRD1-S may primarily regulate gene expression by binding in close proximity to the TSSs of genes and by mediating acetylation of histones, in particular acetylation of histone 3 lysine 14, and chromatin remodeling through its interactions with MYST2 [7] and PBRM1, respectively.

### The BRD1 interaction networks and their relation to mental disorders

Several studies have implicated *14-3-3* genes with schizophrenia and bipolar disorder [52–55, 68], YWHAE and YWHAZ interact with disrupted in schizophrenia 1 (DISC1) [69], and *Ywhae*^+/–^ and *Ywhaz*^–/–^ mice have been shown to display neurodevelopmental and schizophrenia-associated phenotypes [70, 71]. Furthermore, the *PBRM1* gene has been associated with susceptibility to schizophrenia and bipolar disorder [4, 30, 56–58, 72, 73]. Our finding that these proteins all interact with BRD1 provides a novel line of evidence implicating the network of BRD1, PBRM1, and 14-3-3 proteins with schizophrenia and bipolar disorder.

In addition to the similarities and differences of the molecular and cellular functions of BRD1-S and BRD1-L, we explored their interaction networks for enrichment of disease risk. We observed that the entire BRD1 network is enriched for schizophrenia risk and we found that the BRD1-S network was generally more enriched with schizophrenia risk and perhaps other mental disorders compared to BRD1-L. Moreover, the entire BRD1 network was more enriched with schizophrenia compared to the BRD1-S network, suggesting an enhanced effect of combining the networks from both isoforms. Our results support a recently published large-scale study of autism risk genes which demonstrates the importance of considering splice isoforms when exploring disease networks [74]. It should be noted that the identified BRD1 networks were not identified in brain-derived cells. However, our integrative analysis using spatiotemporal expression data from the human brain suggest that the identified interactions may also be occurring and operating in the human brain.

Although the BRD1 interaction network was not identified to be enriched with bipolar disorder risk genes, it is reasonable to speculate that this difference is based on power constraints in the original GWAS rather than on a less prominent role of BRD1 in the etiology of bipolar disorder. Future studies in larger bipolar disorder GWAS datasets are warranted to confirm this hypothesis. In general, the smaller GWAS sets may be underpowered which should be taken into consideration when interpreting the results.

Further integrative analyses using spatiotemporal transcriptome data from the human brain suggested that both BRD1-S and BRD1-L interaction networks play a role in brain function and schizophrenia, especially at mid-fetal stages (pcw 16–26) in the hippocampus, amygdala, and striatum. However, the BRD1-S networks seem also to play a role in brain function and schizophrenia later in life, particularly during childhood and early adulthood. These observations are supportive of the enrichment analysis showing the highest enrichment of schizophrenia risk when analyzing the entire BRD1 network as well as more enrichment of schizophrenia risk when analyzing the BRD1-S network compared to the BRD1-L network.

## Conclusions

Here we expand the molecular and functional characterization of BRD1 and provide evidence that BRD1 acts as a regulatory hub in a comprehensive schizophrenia risk network and possibly risk networks for other mental disorders as well, thereby supporting previous association studies implicating the *BRD1* gene with schizophrenia and bipolar disorder [1–6]. Furthermore, we identify spatiotemporal intervals in the human brain where BRD1 sub-networks are likely to play a role in brain function and schizophrenia. These results encourage further research of BRD1, for example using in vivo models.

## Additional files

Additional file 1: Immunofluorescence staining of HEK293T cells stably expressing V5-epitope tagged BRD1-S and BRD1-L. Stable cell lines (BRD1-S-V5 and BRD1-L-V5) and the untransfected HEK293T cell line (HEK293T) were cultured for 48 h in 2 mL slide flasks followed by fixation in 2 % freshly prepared paraformaldehyde solution. Slides were incubated with anti-V5 antibody (Invitrogen), washed, and incubated with anti-mouse immunoglobulins/FITC F(ab’)2. Cell nuclei were stained by Hoechst staining. From the left: immunoflourescence staining of cell nuclei (Hoechst, *blue*), imunnoflourescence staining of V5-tagged BRD1-S and BRD1-L (Anti-V5, *green*), and the two images merged (Merge, *purple*). (PDF 94 kb) Additional file 2: Expression arrays from siRNA knockdown and over-expression. After RNA purification from BRD1-S, BRD1-L and HEK293T cells, and cells treated with siRNA directed against BRD1 and cells treated with scrambled siRNA, expression microarray analyses were performed on Affymentrix U219 chips. Individual probe emission intensities were normalized to the mean intensity of the entire array and differential values were calculated as log_2_(sample) – log_2_(control) and a cutoff value of +/– 1.5-fold determined if genes were considered upregulated or downregulated. The figure shows *MA-plots*, where M = log_2_(sample) – log_2_(control) and A = ½ × (log_2_(sample) – log_2_(control). The two *horizontal lines* represent 1.5 in expression fold change compared to controls. Probes regulated more than 1.5-fold are indicated by *red dots*. Probes that are not regulated more than 1.5-fold are colored *blue*. MA-plots from the top: (**A**) BRD1-S-V5 stable cells vs. control, BRD1-L-V5 stable cells vs. control and (**B**) cells treated with siRNA directed against BRD1 vs. cells treated with scrambled siRNA (2 array for method 1, M1.1, and M1.2, and 1 for method 2, M2), and control vs. control (ctrl_v_ctrl, from HEK293T cells treated with scrambled siRNA). The *MA-plot* of two independent siRNA knockdown control experiments (ctrl_v_ctrl) showed 0.5 % of the probes below or above a 1.5-fold threshold from a total of 49,386 probes. On average, the analysis showed 4.4 % of the probes to be within threshold limits. (PDF 225 kb) Additional file 3: Immunoprecipitation of PBRM1 and identification of BRD1-S and BRD1-L by western blotting. Co-immunoprecipitations of endogenous PBRM1 were performed on BRD1-S, BRD1-L, and untransfected HEK293T cells, by either including antibody against PBRM1 (AB +) or by not including antibody (AB –). BRD1-S-V5 and BRD1-L-V5 were visualized on the western blot using anti-V5 antibody (Invitrogen) and HRP-conjugated anti-mouse IgG antibody. Crude cell extracts (*E*) from BRD1-S-V5 and BRD1-L-V5 were included as migration markers of the epitope tagged proteins, at approximately 130 kDa and 140 kDa, respectively. (PDF 122 kb) Additional file 4: Supporting information for ChIP-seq analysis. **A** Fragmented DNA for ChIP-seq, with an average size of 100–150 bp from HEK293T cells and stable BRD1-S-V5 and BRD1-L-V5 expressing HEK293T cells. **B** IP-western blot of IPed BRD1-S-V5 and BRD1-L-V5 using V5-ChIP-protocol. V5-tagged BRD1-S and BRD1-L was IPed from stable expressing HEK293T cells using anti-V5 antibody conjugated agarose beads (IP-V5) or anti-HA antibody conjugated beads (IP-HA) as control IP. V5-tagged BRD1-S and BRD1-L were visualized on a western blot using an anti-V5 antibody (Invitrogen) and a HRP-conjugated anti-mouse IgG. (PDF 78 kb) Additional file 5: ChIP-seq mapped reads. This file contains the number of mapped reads for all ChIPseq analyses. (PDF 21 kb) Additional file 6: Chromatin binding profiles for BRD1-S and BRD1-L in relation to identified epigenetic marks in HEK293 cell lines. ChIP-seq data (fastq files), from HEK293 cell lines and cell line derivatives, were obtained from the ENCODE project and mapped to hg19 (for further details see Methods). An in-house histone H3K9ac ChIP-seq dataset was also included in the analysis. The minimum distance from (**A**) BRD1-S or (**B**) BRD1-L ChIP-seq peak to transcription factor binding site or histone mark was identified for all 2205 and 1722 peaks, respectively. The results were summarized as *histograms* with a +/– 30 kb window from BRD1 binding (distance from BRD1 peaks). The number of peaks within each of the summed bins (*columns*) can be read on the *y-axis* (number of peaks). Random ChIP-seq peak regions (Random), with the same composition of chromosomes and peak region sizes, were generated to evaluate if the results could be explained be chance. (PDF 635 kb) Additional file 7: Primers used in ChIP-QPCR and mRNA expression analyses. This file contains primer sequences used for ChIP-QPCR verification of ChIPseq peaks located in the promoter regions of five selected genes. (PDF 21 kb) Additional file 8: Verification of five selected ChIP-seq promoter targets by ChIP-QPCR. From the 251 common promotor target genes (PTGs) of BRD1-S and BRD1-L, we selected five for validation with ChIP-QPCR. **A** The promoter loci at the WD repeat domain 7 (WDR7), digestive organ expansion factor homolog (C1ORF107 or DIEXF), proteasome subunit β type, 2 (PSMB2), ZC3H15, and zinc finger protein 226 (ZNF226) showed BRD1-S and BRD1-L binding, while the promoter loci of the lymphocyte expressed gene granzyme M (GZMM) showed no significant binding of the BRD1 isoforms and was subsequently selected as a negative control. The BRD1-S or BRD1-L binding is shown in *black* whereas the control ChIP-seq (IP with anti-HA antibody) is shown in *pink*. **B** Primers were designed to amplify a 80–150 bp sequence in the promoter region or the intron of all six genes (for primer sequences see Additional file 7). ChIP was performed with either anti-V5 antibody (V5) conjugated beads or anti-HA (HA) conjugated beads using extracts from BRD1-S, BRD1-L, or HEK293T cells (for further details see “Methods”). ChIP-QPCR showed higher enrichment of BRD1-S and BRD1-L at the promoter loci of WDR7, DIEXF, PSMB2, ZC3H15, and ZNF226 compared to controls. Also ChIP-QPCR showed less BRD1-S and BRD1-L binding at introns of WDR7, DIEXF, PSMB2, ZC3H15, and ZNF226 while ChIP-QPCR showed lower and approximately equal BRD1-S and BRD1-L binding at the GZMM promoter and intron. (PDF 433 kb) Additional file 9: Ingenuity pathway analysis of BRD1 interaction networks. This Excel file includes three spreadsheets containing enriched categories, sub-categories, and canonical pathways identified by Ingenuity pathway analysis on the BRD1 interaction network. (XLSX 34 kb) Additional file 10: Upregulation of BRD1-S and BRD1-L. **A** QPCR measurement of BRD1 mRNA in BRD1-S and BRD1-L cells showed a ~24-fold higher BRD1 mRNA levels compared to HEK293T controls (**P* <0.0001). *Error bars* indicate the standard error of the mean from n = 2 independent RNA samples. **B** *Western blotting analysis* using an antibody against endogenous BRD1 (BRD1 S/L) showed high levels of BRD1-S and BRD1-L in the stable cell lines compared to the expression level of BRD1 in HEK293T cells (293 T). **D** Expression array data showing log2 fold change values for probes that target BRD1 and seven housekeeping genes: Glyceraldehyde 3-phosphate dehydrogenase (GAPDH), Ubiquitin C (UBC), TATA box binding protein (TBP), hypoxanthine phosphoribosyltransferase 1 (HPRT1), Beta Actin (ACTB), Beta-2 microglobulin (B2M), and glucuronidase beta (GUSB). (PDF 76 kb) Additional file 11: Knockdown of endogenous BRD1 using pools of small interfering RNAs (siRNAs). In order to downregulate the endogenous BRD1 level, (**A**) HEK293T cells were treated with two different pools of four different siRNA directed against BRD1 (BRD1 siRNA) and scrambled siRNA (Ctrl siRNA) as a control. Kits from Dharmacon (Method 1) and Qiagen (Method 2) were used at concentrations of 12.5 and 5.5 nM, respectively, and cells were harvested 24 h after transfection followed by QPCR measurement of BRD1 mRNA. BRD1 mRNA levels were significantly lower (approximately 70–80 % reduction for method 1 and 60 % for method 2) in cells treated with BRD1 directed siRNA compared to cells treated with scrambled siRNA (****P* <0.0001). The knockdown experiments showing 80 %, 70 %, and 60 % decreased levels of BRD1 mRNA are referred to as KD1, KD2, and KD3, respectively. Error bars indicate the standard error of the mean (SEM) from n = 3 independent RNA samples. **B** Expression array data showing log2 fold change values for probes that target BRD1 and seven housekeeping genes: Glyceraldehyde 3-phosphate dehydrogenase (GAPDH), Ubiquitin C (UBC), TATA box binding protein (TBP), hypoxanthine phosphoribosyltransferase 1 (HPRT1), Beta Actin (ACTB), Beta-2 microglobulin (B2M), and glucuronidase beta (GUSB). **C** *Western blot* of siRNA silencing of endogenous BRD1 in HEK293T cells using method 1 and 2. BRD1 directed siRNA silencing and control transfections with scrambled siRNA (Ctrl siRNA/Unspecific siRNA) was performed at increasing concentrations. The starting concentrations are approximately 50 % of the manufacturer’s recommended concentrations. **D** *Photometric analysis* of western blot in (**B**), showing decreased levels of BRD1 in cells treated with BRD1 directed siRNA. The BRD1 band intensities are normalized to beta-tubulin intensities and are shown as relative to the highest value. (PDF 165 kb) Additional file 12: Metadata and statistics of integrative ChIP-seq and expression analysis. This file contains three Excel spreadsheets. Spreadsheet 1 contains information on the number of genes common to both the exon expression array and ChIPseq analyses. Spreadsheets 2 and 3 include statistical and permutation tests concerning enrichment of DEGs among BRD1 PTGs. (XLSX 12 kb) Additional file 13: Integrative analysis of ChIP-seq and gene-expression data. In order to investigate the gene-regulatory potential of BRD1-S and BRD1-L, ChIPseq data were integrated with expression data. A window of +/– 200 kb from BRD1 binding was constructed, fragmented in 9 kb segments, and determined the number of DEGs having TSSs in these segments. As opposed to the 10 kb segment used in the main manuscript the 9 kb segments used here span across 0 on the x-axis. **A**, **B** The analysis was performed for DEGs identified after upregulating BRD1-S or BRD1-L or (**C**, **D**) downregulating endogenous BRD1. Significantly more up- or downregulated genes were identified in segments where BRD1-S or BRD1-L binds in close proximity to TSSs compared to all segments across the window (*P* <0.001, 1-sample Student’s *t*-test). **E**, **F** The analysis was also performed after upregulating BRD1-S or BRD1-L and using a narrow window of +/– 5 kb from BRD1 binding sites. In order to investigate the BRD1 gene-regulatory potential while considering all genes on the array (and not only DEGs), probes were segmented according to TSS distance from BRD1 binding site following calculation of the average fold change of all probes within windows +/– 10 kb and +/– 200 kb from BRD1binding sites. **F**, **G** These two analyses show the mean expression fold changes combined from several genes and their distance from either BRD1-S or BRD1-L binding on the genome. **F** The first analysis shows the mean probe fold change in segments of 400 probes and using a +/– 10 kb window. **G** The second analysis shows the mean probe fold change in segments of 500 probes and using a +/– 200 kb window. (PDF 753 kb) Additional file 14: Ingenuity pathway analysis of DEGs after overexpressing either BRD1-S or BRD1-L. This Excel spreadsheet contains a list of enriched canonical pathways identified by Ingenuity pathway analysis using the identified DEGs after upregulating either BRD1-S or BRD1-L. (XLSX 16 kb) Additional file 15: Spatiotemporal mRNA expression of BRD1 in human brain. **A** RNA-seq data (obtained from Brainspan; http://www.brainspan.org) showing the temporal expression of BRD1 across 26 brain regions; primary auditory cortex, core (*A1C*), amygdaloid complex (*AMY*), cerebellar cortex (*CBC*), cerebellum (CB), caudal ganglionic eminence (*CGE*), dorsolateral prefrontal cortex (*DFC*), dorsal thalamus (*DTH*), hippocampus (hippocampal formation) (*HIP*), posteroventral (inferior) parietal cortex (*IPC*), inferolateral temporal cortex (area TEv) (*ITC*), lateral ganglionic eminence (*LGE*), primary motor cortex (area M1) (*M1C*), primary motor-sensory cortex (samples) (*M1C-S1C*), mediodorsal nucleus of thalamus (*MD*), anterior (rostral) cingulate (medial prefrontal) cortex (*MFC*), medial ganglionic eminence (*MGE*), occipital neocortex (*Ocx*), orbital frontal cortex (*OFC*), parietal neocortex (*PCx*), primary somatosensory cortex (area S1) (*S1C*), posterior (caudal) superior temporal cortex (area 22c) (*STC*), striatum (*STR*), temporal neocortex (*TCx*), upper (rostral) rhombic lip (*URL*), primary visual cortex (striate cortex) (*V1C*), ventrolateral prefrontal cortex (*VFC*). Age on the *x-axis* is shown as log10(days) (log10(age)) and the gene expression is shown as the average reads per kilobase per million (avg_rpkm). **B** Expression microarray data (obtained from the Human Brain Transcriptome, HBT; http://hbatlas.org) showing the temporal expression of BRD1 across six brain regions: neocortex (*NCX*), hippocampus (*HIP*), amygdala (*AMY*), striatum (*STR*), mediodorsal nucleus of thalamus (*MD*), cerebellar cortex (*CBC*). Both datasets show high expression of BRD1 in early fetal stages (until approximately day 180) and both datasets show high expression of BRD1 in the cerebellar cortex throughout the timeline of the datasets. (PDF 208 kb) Additional file 16: Supporting information for the gene-set enrichment analyses using MAGMA. This file contains two Excel spreadsheets. Spreadsheet 1 contains gene-wide and experiment-wide significant genes from the gene-set enrichment analysis of the BRD1 interaction network and schizophrenia. Spreadsheet 2 contains all BRD1 gene sets used in the analysis. (XLSX 45 kb) Additional file 17: Spatiotemporal co-expression landscapes of BRD1-S and BRD1-L PPIs and PTGs in the human brain. The fraction of co-expressed genes with BRD1 across 32 spatiotemporal intervals. **A** The fraction of genes co-expressed with BRD1 across all genes (*gray*) and genes in the BRD1-S and BRD1-L PPI networks (*red* and *orange*). None of these were significant at *P* <0.05 after adjusting for multiple tests. (#) denotes *P* <0.05 before correcting for multiple testing. **B** The fraction of genes co-expressed with BRD1 across all genes (*gray*) and genes in the BRD1-S and BRD1-L PTGs + PPI networks (*red* and *orange*). A one-sided binominal test was performed for each BRD1 sub-network and spatiotemporal interval compared to the expected fraction in the background where the asterisk (*) denotes *P* <0.05 after adjusting for multiple tests. (PDF 322 kb)

## Abbreviations

BRD1: Bromodomain containing 1
DEG: Differentially expressed gene
IPA: Ingenuity pathway analysis
PBAF: Polybromo-associated BRG1 associated factor
PPI: Protein-protein interaction
PTG: Promotor target gene
SWI/SNF: SWItch/Sucrose Non-Fermentable
TSS: Transcription start site
UTR: Untranslated region
