# Supplementary material for: Identification of the BRD1 interaction network and its impact on mental disorder risk
Source: Genome Med. 2016 May 3;8:53. doi: 10.1186/s13073-016-0308-x (PMC4855718; doi:10.1186/s13073-016-0308-x)
Supplement: Additional file 1: — Immunofluorescence staining of HEK293T cells stably expressing V5-epitope tagged BRD1-S and BRD1-L. Stable cell lines (BRD1-S-V5 and BRD1-L-V5) and the untransfected HEK293T cell line (HEK293T) were cultured for 48 h in 2 mL slide flasks followed by fixation in 2 % freshly prepared paraformaldehyde solution. Slides were incubated with anti-V5 antibody (Invitrogen), washed, and incubated with anti-mouse immunoglobulins/FITC F(ab’)2. Cell nuclei were stained by Hoechst staining. From the left: immunoflourescence staining of cell nuclei (Hoechst, blue), imunnoflourescence staining of V5-tagged BRD1-S and BRD1-L (Anti-V5, green), and the two images merged (Merge, purple). (PDF 94 kb) [file 13073_2016_308_MOESM1_ESM.pdf]

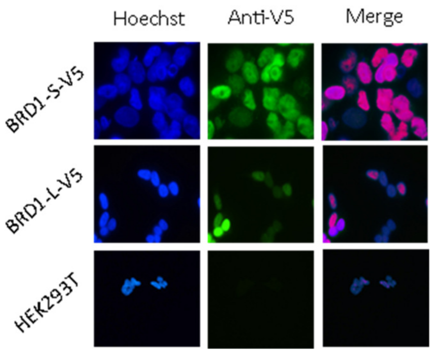

Immunofluorescence staining of HEK293T cells stably expressing V5-epitope tagged BRD1-S and BRD1-L. Stable cell lines (BRD1-S-V5 and BRD1-L-V5) and the untransfected HEK293T cell line (HEK293T) were cultured for 48 hours in 2 mL slide flasks followed by fixation in 2% freshly prepared paraformaldehyde solution. Slides were incubated with anti-V5 antibody (Invitrogen), washed, and incubated with anti-mouse immunoglobulins /FITC F(ab')2. Cell nuclei were stained by Hoechst staining (for further details see the full materials and methods above). From the left: immunoflourescence staining of cell nuclei (Hoechst, blue), imunnoflourescence staining of V5-tagged BRD1-S and BRD1-L (Anti-V5, green), and the two images merged (Merge, purple).
