# Supplementary material for: Identification of the BRD1 interaction network and its impact on mental disorder risk
Source: Genome Med. 2016 May 3;8:53. doi: 10.1186/s13073-016-0308-x (PMC4855718; doi:10.1186/s13073-016-0308-x)
Supplement: Additional file 2: — Expression arrays from siRNA knockdown and over-expression. After RNA purification from BRD1-S, BRD1-L and HEK293T cells, and cells treated with siRNA directed against BRD1 and cells treated with scrambled siRNA, expression microarray analyses were performed on Affymentrix U219 chips. Individual probe emission intensities were normalized to the mean intensity of the entire array and differential values were calculated as log2(sample) – log2(control) and a cutoff value of +/– 1.5-fold determined if genes were considered upregulated or downregulated. The figure shows MA-plots, where M = log2(sample) – log2(control) and A = ½ × (log2(sample) – log2(control). The two horizontal lines represent 1.5 in expression fold change compared to controls. Probes regulated more than 1.5-fold are indicated by red dots. Probes that are not regulated more than 1.5-fold are colored blue. MA-plots from the top: (A) BRD1-S-V5 stable cells vs. control, BRD1-L-V5 stable cells vs. control and (B) cells treated with siRNA directed against BRD1 vs. cells treated with scrambled siRNA (2 array for method 1, M1.1, and M1.2, and 1 for method 2, M2), and control vs. control (ctrl_v_ctrl, from HEK293T cells treated with scrambled siRNA). The MA-plot of two independent siRNA knockdown control experiments (ctrl_v_ctrl) showed 0.5 % of the probes below or above a 1.5-fold threshold from a total of 49,386 probes. On average, the analysis showed 4.4 % of the probes to be within threshold limits. (PDF 225 kb) [file 13073_2016_308_MOESM2_ESM.pdf]

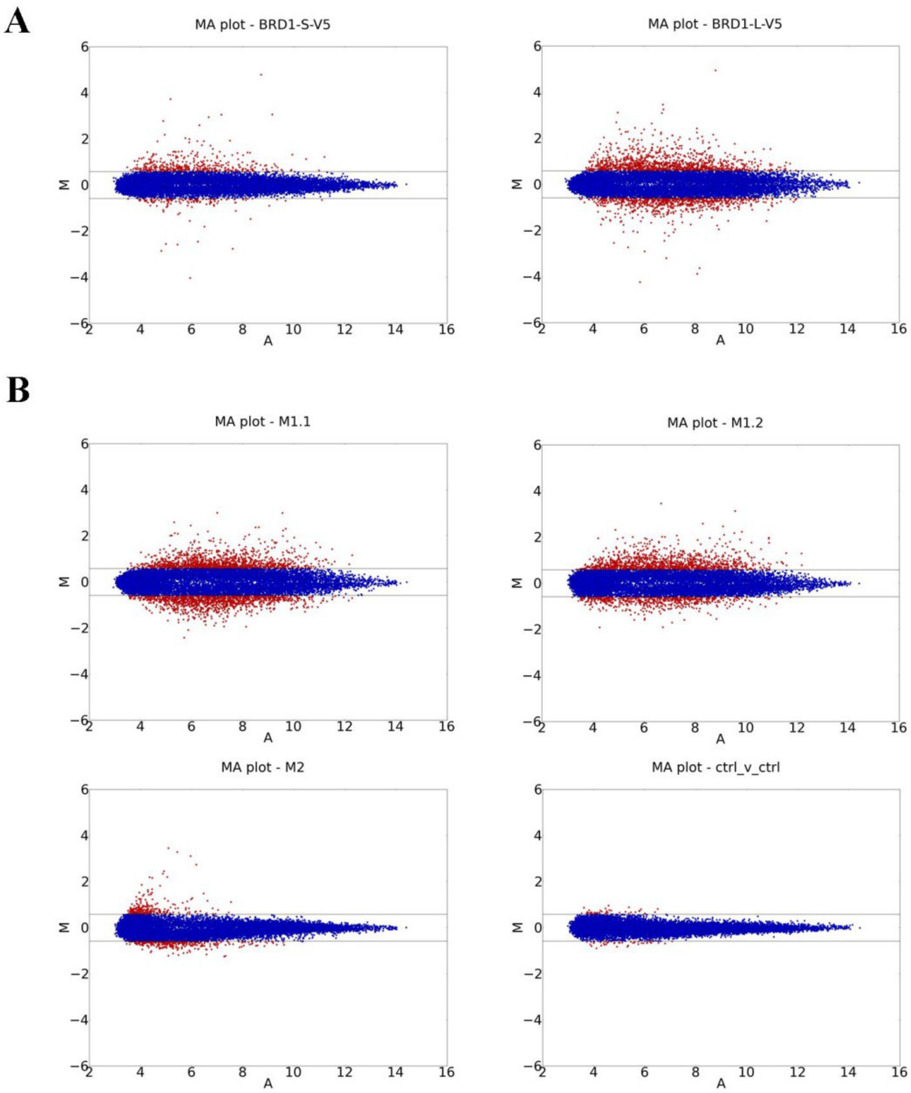
A
MA plot - BRD1-S-V5
MA plot - BRD1-L-V5
B
MA plot - M1.1
MA plot - M1.2
MA plot - M2
MA plot - ctrl_v_ctrl
M
A

Expression arrays from siRNA knockdown and over-expression. After RNA purification from BRD1-S, BRD1-L and HEK293T cells, and cells treated siRNA directed against *BRD1* and cells treated with scrambled siRNA, expression microarray analyses were performed on Affymentrix U219 chips. Individual probe emission intensities were normalized to the mean intensity of the entire array and differential values were calculated as $\log_2$(sample) – $\log_2$(control) and a cut-off value of +/- 1.5 fold determined if genes were considered up-regulated or down-regulated. The figure shows MA-plots, where M = $\log_2$(sample) – $\log_2$(control) and A = ½ x ($\log_2$(sample) - $\log_2$(control). The two horizontal lines represent 1.5 in expression fold change compared to controls. Probes regulated more than 1.5 fold are indicated by red dots. Probes that are not regulated more than 1.5 fold are colored blue. MA-plots from the top: (A) BRD1-S-V5 stable cells vs control, BRD1-L-V5 stable cells vs control and (B) cells treated with siRNA directed against *BRD1* vs cells treated with scrambled siRNA (2 array for method 1, M1.1 and M1.2, and 1 for method 2, M2), and control vs control (ctrl_v_ctrl, from HEK293T cells treated with scrambled siRNA). The MA-plot of two independent siRNA knockdown control experiments (ctrl_v_ctrl) showed 0.5% of the probes below or above a 1.5 fold threshold from a total of 49.386 probes. On average, the analysis showed 4.4% of the probes to be within threshold limits.
