# Supplementary material for: Identification of the BRD1 interaction network and its impact on mental disorder risk
Source: Genome Med. 2016 May 3;8:53. doi: 10.1186/s13073-016-0308-x (PMC4855718; doi:10.1186/s13073-016-0308-x)
Supplement: Additional file 3: — Immunoprecipitation of PBRM1 and identification of BRD1-S and BRD1-L by western blotting. Co-immunoprecipitations of endogenous PBRM1 were performed on BRD1-S, BRD1-L, and untransfected HEK293T cells, by either including antibody against PBRM1 (AB +) or by not including antibody (AB –). BRD1-S-V5 and BRD1-L-V5 were visualized on the western blot using anti-V5 antibody (Invitrogen) and HRP-conjugated anti-mouse IgG antibody. Crude cell extracts (E) from BRD1-S-V5 and BRD1-L-V5 were included as migration markers of the epitope tagged proteins, at approximately 130 kDa and 140 kDa, respectively. (PDF 122 kb) [file 13073_2016_308_MOESM3_ESM.pdf]

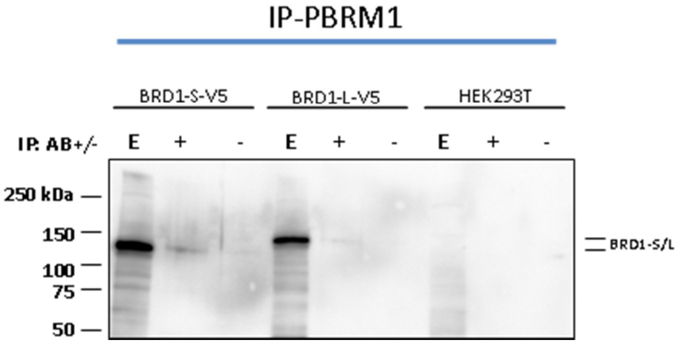

Immunoprecipitation of PBRM1 and identification of BRD1-S and BRD1-L by western blotting. Co-immunoprecipitations of endogenous PBRM1 were performed on BRD1-S, BRD1-L and untransfected HEK293T cells, by either including antibody against PBRM1 (AB +) or by not including antibody (AB -). BRD1-S-V5 and BRD1-L-V5 were visualized on the western blot using anti-V5 antibody (Invitrogen) and HRP- conjugated anti-mouse IgG antibody (for further details see the full materials and methods above). Crude cell extracts (E) from BRD1-S-V5 and BRD1-L-V5 were included as migration markers of the epitope tagged proteins, at approximately 130 kDa and 140 kDa, respectively.
