# Supplementary material for: Identification of the BRD1 interaction network and its impact on mental disorder risk
Source: Genome Med. 2016 May 3;8:53. doi: 10.1186/s13073-016-0308-x (PMC4855718; doi:10.1186/s13073-016-0308-x)
Supplement: Additional file 4: — Supporting information for ChIP-seq analysis. A Fragmented DNA for ChIP-seq, with an average size of 100–150 bp from HEK293T cells and stable BRD1-S-V5 and BRD1-L-V5 expressing HEK293T cells. B IP-western blot of IPed BRD1-S-V5 and BRD1-L-V5 using V5-ChIP-protocol. V5-tagged BRD1-S and BRD1-L was IPed from stable expressing HEK293T cells using anti-V5 antibody conjugated agarose beads (IP-V5) or anti-HA antibody conjugated beads (IP-HA) as control IP. V5-tagged BRD1-S and BRD1-L were visualized on a western blot using an anti-V5 antibody (Invitrogen) and a HRP-conjugated anti-mouse IgG. (PDF 78 kb) [file 13073_2016_308_MOESM4_ESM.pdf]

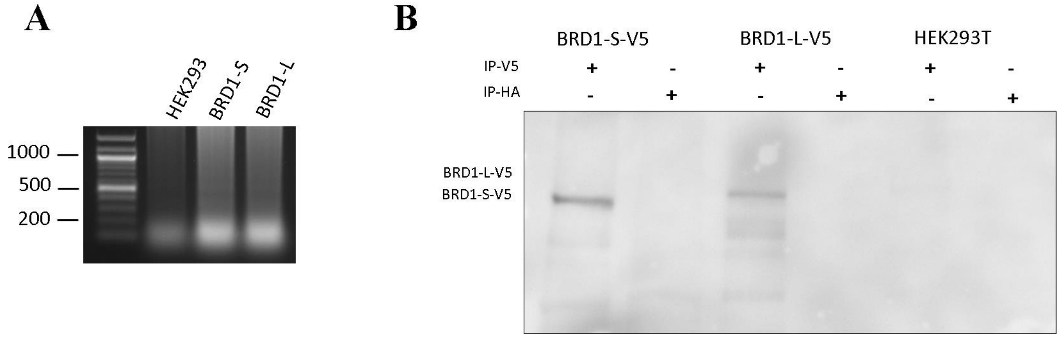

ChIP-seq analysis. (A) Fragmented DNA for ChIP-seq, with an average size of 100-150 bp from HEK293T cells and stable BRD1-S-V5 and BRD1-L-V5 expressing HEK293T cells. (B) IP-Western blot of IPed BRD1-S-V5 and BRD1-L-V5 using V5-ChIP-protocol. V5-tagged BRD1-S and BRD1-L was IPed from stable expressing HEK293T cells using anti-V5 antibody conjugated agarose beads (IP-V5) or anti-HA antibody conjugated beads (IP-HA) as control IP. V5-tagged BRD1-S and BRD1-L were visualized on a western blot using an anti-V5 antibody (Invitrogen) and a HRP conjugated anti-mouse IgG.
