# Supplementary material for: Identification of the BRD1 interaction network and its impact on mental disorder risk
Source: Genome Med. 2016 May 3;8:53. doi: 10.1186/s13073-016-0308-x (PMC4855718; doi:10.1186/s13073-016-0308-x)
Supplement: Additional file 5: — ChIP-seq mapped reads. This file contains the number of mapped reads for all ChIPseq analyses. (PDF 21 kb) [file 13073_2016_308_MOESM5_ESM.pdf]

ChIP-seq mapped reads*

ChIP-seq experiment	*Number of mapped reads*
BRD1-L-V5 Anti-V5 sample	29,948,443
BRD1-L-V5 Anti-HA control	16,403,259
BRD1-S-V5 Anti-V5 sample	27,999,799
BRD1-S-V5 Anti-HA control	24,085,129
Average	24,609,158

*NGS performed using the Genome Analyzer IIx. Reads were mapped using Bowtie allowing 1 mismatch
