# Supplementary material for: Identification of the BRD1 interaction network and its impact on mental disorder risk
Source: Genome Med. 2016 May 3;8:53. doi: 10.1186/s13073-016-0308-x (PMC4855718; doi:10.1186/s13073-016-0308-x)
Supplement: Additional file 6: — Chromatin binding profiles for BRD1-S and BRD1-L in relation to identified epigenetic marks in HEK293 cell lines. ChIP-seq data (fastq files), from HEK293 cell lines and cell line derivatives, were obtained from the ENCODE project and mapped to hg19 (for further details see Methods). An in-house histone H3K9ac ChIP-seq dataset was also included in the analysis. The minimum distance from (A) BRD1-S or (B) BRD1-L ChIP-seq peak to transcription factor binding site or histone mark was identified for all 2205 and 1722 peaks, respectively. The results were summarized as histograms with a +/– 30 kb window from BRD1 binding (distance from BRD1 peaks). The number of peaks within each of the summed bins (columns) can be read on the y-axis (number of peaks). Random ChIP-seq peak regions (Random), with the same composition of chromosomes and peak region sizes, were generated to evaluate if the results could be explained be chance. (PDF 635 kb) [file 13073_2016_308_MOESM6_ESM.pdf]

A

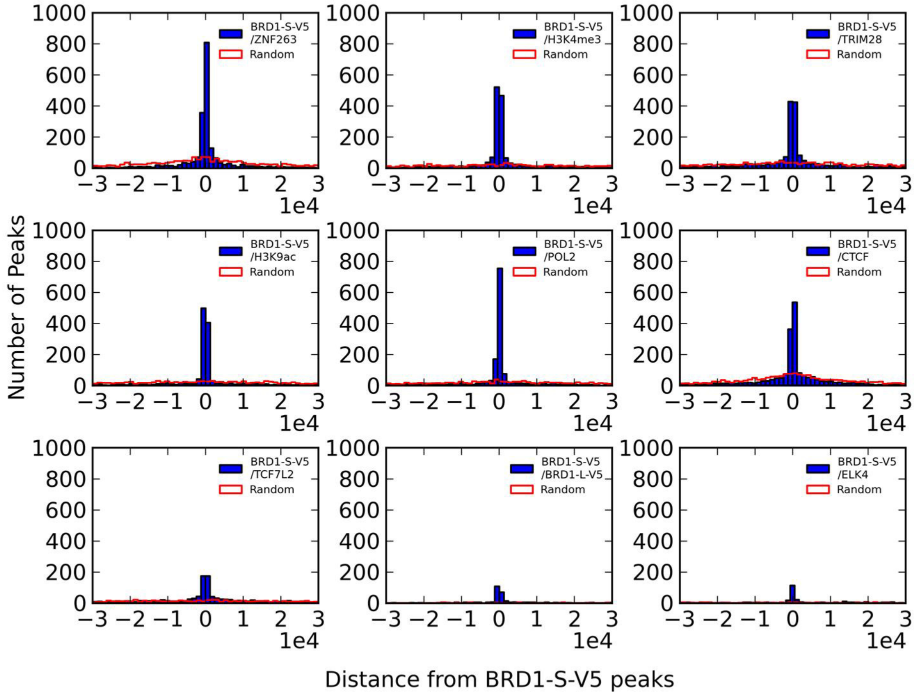

BRD1-S-V5 /ZNF263
BRD1-S-V5 /H3K4me3
BRD1-S-V5 /TRIM28
BRD1-S-V5 /H3K9ac
BRD1-S-V5 /POL2
BRD1-S-V5 /CTCF
BRD1-S-V5 /TCF7L2
BRD1-S-V5 /BRD1-L-V5
BRD1-S-V5 /ELK4
Random
Number of Peaks
1e4
Distance from BRD1-S-V5 peaks

B

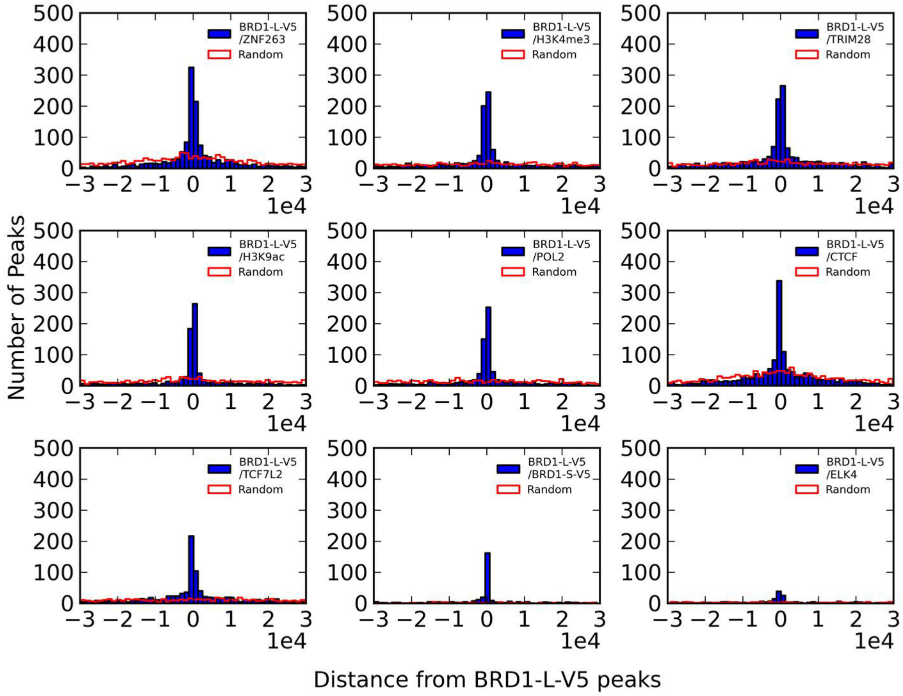

BRD1-L-V5 /ZNF263
BRD1-L-V5 /H3K4me3
BRD1-L-V5 /TRIM28
BRD1-L-V5 /H3K9ac
BRD1-L-V5 /POL2
BRD1-L-V5 /CTCF
BRD1-L-V5 /TCF7L2
BRD1-L-V5 /BRD1-S-V5
BRD1-L-V5 /ELK4
Random
Number of Peaks
1e4
Distance from BRD1-L-V5 peaks

Chromatin binding profiles for BRD1-S and BRD1-L in relation to identified epigenetic marks in HEK293 cell lines. ChIP-seq data (fastq files), from HEK293 cell lines and cell line derivatives, was obtained from the ENCODE project and mapped to hg19 (for further details see Materials and Methods). An *in house* histone H3K9ac ChIP-seq dataset was also included in the analysis. The minimum distance from (A) BRD1-S or (B) BRD1-L ChIP-seq peak to transcription factor binding site or histone mark was identified for all 2205 and 1722 peaks, respectively. The results were summarized as histograms with a +/- 30 kb window from BRD1 binding (distance from BRD1 peaks). The number of peaks within each of the summed bins (columns) can be read on the y-axis (Number of peaks). Random ChIP-seq peak regions (Random), with the same composition of chromosomes and peak region sizes, were generated to evaluate if the results could be explained be chance.
