# Supplementary material for: Identification of the BRD1 interaction network and its impact on mental disorder risk
Source: Genome Med. 2016 May 3;8:53. doi: 10.1186/s13073-016-0308-x (PMC4855718; doi:10.1186/s13073-016-0308-x)
Supplement: Additional file 7: — Primers used in ChIP-QPCR and mRNA expression analyses. This file contains primer sequences used for ChIP-QPCR verification of ChIPseq peaks located in the promoter regions of five selected genes. (PDF 21 kb) [file 13073_2016_308_MOESM7_ESM.pdf]

Primers used in ChIP-QPCR and mRNA expression analyses

Number	*Gene*	*Location*	*Primer sequence 5' - 3'*
1	*ZNF226*	promoter	TGTTCTGTTTTTCCCCCAAC
2	*ZNF226*	promoter	CACGGAACTCCGGACTACAT
3	*ZNF226*	intron	GCCATCACCAGGAACATACC
4	*ZNF226*	intron	TTGGATGCAGCAAGCTAAAA
5	*DIEXF*	promoter	TGAGAACACTAGTGAACGAACTCC
6	*DIEXF*	promoter	CCCTACCGCAGCTCTTTGAA
7	*DIEXF*	intron	GACCCTTTTTCCCCCAGTTA
8	*DIEXF*	intron	AGGATGTTCAAAGCAGCACT
9	*WDR7*	promoter	CTCCACTTTCGGTCTCCATC
10	*WDR7*	promoter	TCCCTACTTGCAAGGCCTAA
11	*WDR7*	intron	TTGGCTATATGGTTGTATTTTGGA
12	*WDR7*	intron	CACAGCACTGGGGAAAGATT
13	*PMSB2*	promoter	GTGCTTGTCTCTGGGATCGT
14	*PMSB2*	promoter	AAACTGGGCGTCACATAAGG
15	*PMSB2*	intron	TCTCCTCTTGCCTTTGCTTC
20	*PMSB2*	intron	CAAATTTAGGGATGGGGACA
21	*ZC3H15*	promoter	CATCTGAAAAGGGCCTATCG
22	*ZC3H15*	promoter	ACGTGGGAAAATGACTACGC
23	*ZC3H15*	intron	GAAGCCTTTTTGAACTTCTCCA
24	*ZC3H15*	intron	GCACCTCTTGAAAACAGAATGA
25	*GZMM*	promoter	GTGGCTTCATCTCGAGTCCT
26	*GZMM*	promoter	ACCCTCCATCCTTGAGCATT
27	*GZMM*	intron	CACAGTCCTGGTGTGGACAG
28	*GZMM*	intron	TCAGCAGGTGAATGGACAGA
29	*BRD1*	exon4/5	ACCAGCTGCAAGACAAGGAC
30	*BRD1*	exon4/5	TTTCCTCATTGTGGCAAAGTC
31	*BRD1*	exon9/10	TGCATCGAGAATGGGAACTAC
32	*BRD1*	exon9/10	ACCACCTTCAGAGGCTCCAG
