# Supplementary material for: Identification of the BRD1 interaction network and its impact on mental disorder risk
Source: Genome Med. 2016 May 3;8:53. doi: 10.1186/s13073-016-0308-x (PMC4855718; doi:10.1186/s13073-016-0308-x)
Supplement: Additional file 8: — Verification of five selected ChIP-seq promoter targets by ChIP-QPCR. From the 251 common promotor target genes (PTGs) of BRD1-S and BRD1-L, we selected five for validation with ChIP-QPCR. A The promoter loci at the WD repeat domain 7 (WDR7), digestive organ expansion factor homolog (C1ORF107 or DIEXF), proteasome subunit β type, 2 (PSMB2), ZC3H15, and zinc finger protein 226 (ZNF226) showed BRD1-S and BRD1-L binding, while the promoter loci of the lymphocyte expressed gene granzyme M (GZMM) showed no significant binding of the BRD1 isoforms and was subsequently selected as a negative control. The BRD1-S or BRD1-L binding is shown in black whereas the control ChIP-seq (IP with anti-HA antibody) is shown in pink. B Primers were designed to amplify a 80–150 bp sequence in the promoter region or the intron of all six genes (for primer sequences see Additional file 7). ChIP was performed with either anti-V5 antibody (V5) conjugated beads or anti-HA (HA) conjugated beads using extracts from BRD1-S, BRD1-L, or HEK293T cells (for further details see “Methods”). ChIP-QPCR showed higher enrichment of BRD1-S and BRD1-L at the promoter loci of WDR7, DIEXF, PSMB2, ZC3H15, and ZNF226 compared to controls. Also ChIP-QPCR showed less BRD1-S and BRD1-L binding at introns of WDR7, DIEXF, PSMB2, ZC3H15, and ZNF226 while ChIP-QPCR showed lower and approximately equal BRD1-S and BRD1-L binding at the GZMM promoter and intron. (PDF 433 kb) [file 13073_2016_308_MOESM8_ESM.pdf]

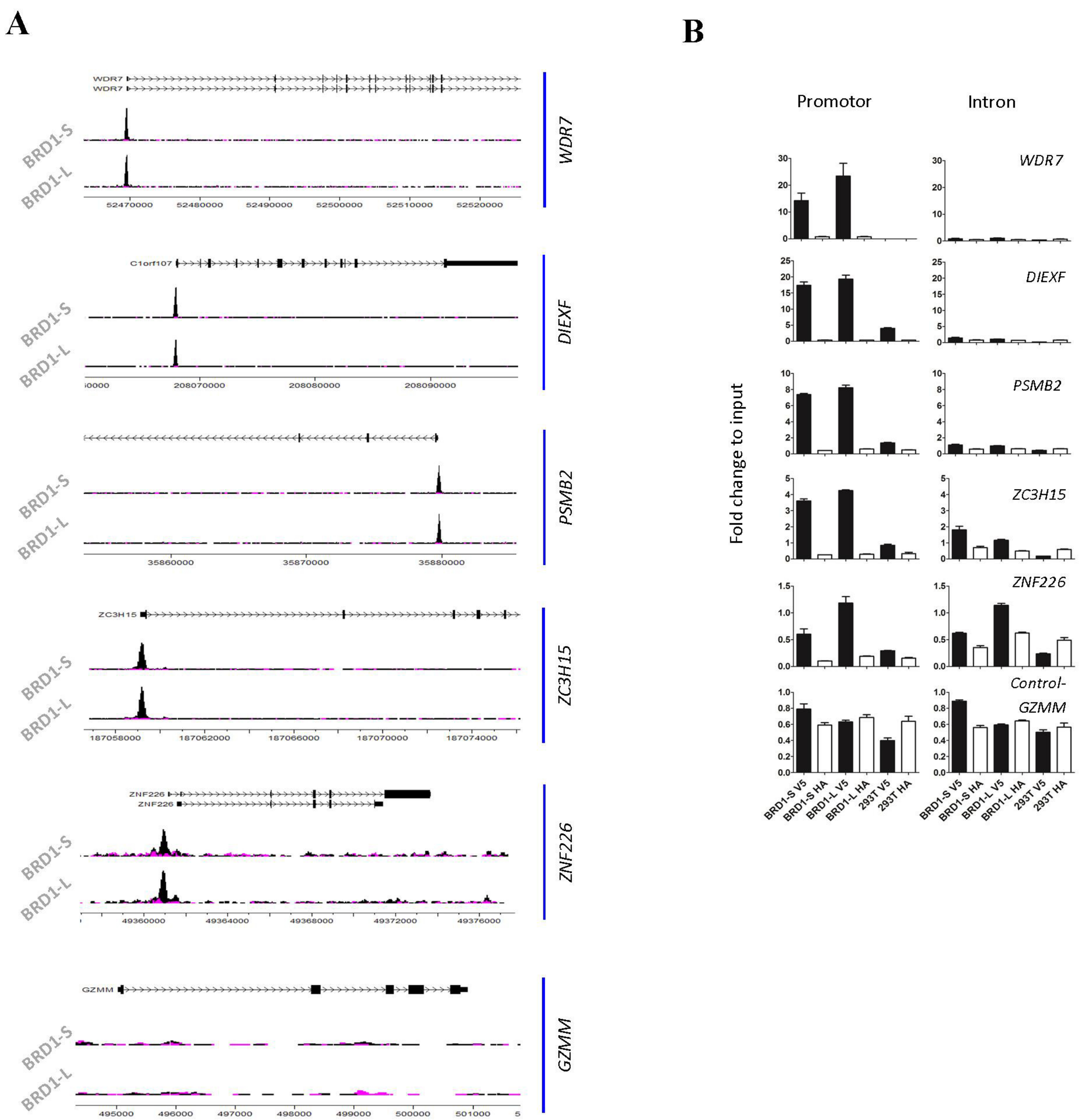
A
B
WDR7
DIEXF
C1orf107
PSMB2
ZC3H15
ZNF226
GZMM
BRD1-S
BRD1-L
Promotor
Intron
Fold change to input
Control-
GZMM
BRD1-S V5
BRD1-S HA
BRD1-L V5
BRD1-L HA
293T V5
293T HA

**Verification of five selected ChIP-seq promoter targets by ChIP-QPCR.** From the 251 common promotor target genes (PTGs) of BRD1-S and BRD1-L, we selected 5 for validation with ChIP-QPCR. (A) The promoter loci at the WD repeat domain 7 (*WDR7*), digestive organ expansion factor homolog (*C1ORF107* or *DIEXF*), proteasome subunit β type, 2 (*PSMB2*), *ZC3H15*, and zinc finger protein 226 (*ZNF226*) showed BRD1-S and BRD1-L binding, while the promoter loci of the lymphocyte expressed gene granzyme M (*GZMM*) showed no significant binding of the BRD1 isoforms and was subsequently selected as a negative control. The BRD1-S or BRD1-L binding is shown in black whereas the control ChIP-seq (IP with anti-HA antibody) is shown in pink. (B) Primers were designed to amplify a 80-150bp sequence in the promoter region or the intron of all 6 genes (for primer sequences see Supplemental Table 2). ChIP was performed with either anti-V5 antibody (V5) conjugated beads or anti-HA (HA) conjugated beads using extracts from BRD1-S, BRD1-L or HEK293T cells (for further details see the materials and methods). ChIP-QPCR showed higher enrichment of BRD1-S and BRD1-L at the promoter loci of *WDR7*, *DIEXF*, *PSMB2*, *ZC3H15*, and *ZNF226* compared to controls. Also ChIP-QPCR showed less BRD1-S and BRD1-L binding at introns of *WDR7*, *DIEXF*, *PSMB2*, *ZC3H15*, and *ZNF226* while ChIP-QPCR showed lower and approximately equal BRD1-S and BRD1-L binding at the *GZMM* promoter and intron.
