# Supplementary material for: Identification of the BRD1 interaction network and its impact on mental disorder risk
Source: Genome Med. 2016 May 3;8:53. doi: 10.1186/s13073-016-0308-x (PMC4855718; doi:10.1186/s13073-016-0308-x)
Supplement: Additional file 10: — Upregulation of BRD1-S and BRD1-L. A QPCR measurement of BRD1 mRNA in BRD1-S and BRD1-L cells showed a ~24-fold higher BRD1 mRNA levels compared to HEK293T controls (*P <0.0001). Error bars indicate the standard error of the mean from n = 2 independent RNA samples. B Western blotting analysis using an antibody against endogenous BRD1 (BRD1 S/L) showed high levels of BRD1-S and BRD1-L in the stable cell lines compared to the expression level of BRD1 in HEK293T cells (293 T). D Expression array data showing log2 fold change values for probes that target BRD1 and seven housekeeping genes: Glyceraldehyde 3-phosphate dehydrogenase (GAPDH), Ubiquitin C (UBC), TATA box binding protein (TBP), hypoxanthine phosphoribosyltransferase 1 (HPRT1), Beta Actin (ACTB), Beta-2 microglobulin (B2M), and glucuronidase beta (GUSB). (PDF 76 kb) [file 13073_2016_308_MOESM10_ESM.pdf]

A

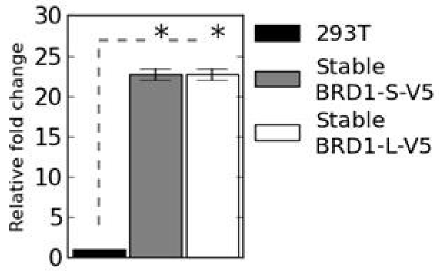

B

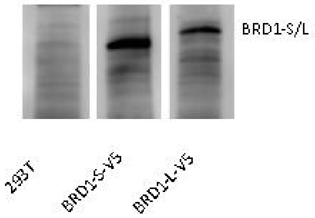

D

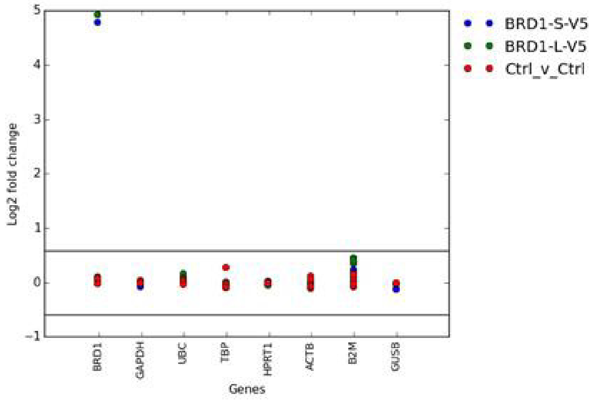

Upregulation of BRD1-S and BRD1-L. (A) QPCR measurement of *BRD1* mRNA in BRD1-S and BRD1-L cells showed a ~24 fold higher *BRD1* mRNA levels compared to HEK293T controls (*$P < 0.0001$). Error bars indicate the standard error of the mean from n = 2 independent RNA samples. (B) Western blotting analysis using an antibody against endogenous BRD1 (BRD1 S/L) showed high levels of BRD1-S and BRD1-L in the stable cell lines compared to the expression level of BRD1 in HEK293T cells (293T). (D) Expression array data showing $\log_2$ fold change values for probes that target *BRD1* and 7 housekeeping genes: Glyceraldehyde 3-phosphate dehydrogenase (*GAPDH*), Ubiquitin C (*UBC*), TATA box binding protein (*TBP*), hypoxanthine phosphoribosyltransferase 1 (*HPRT1*), Beta Actin (*ACTB*), Beta-2 microglobulin (*B2M*), and glucuronidase beta (*GUSB*).
