# Supplementary material for: Identification of the BRD1 interaction network and its impact on mental disorder risk
Source: Genome Med. 2016 May 3;8:53. doi: 10.1186/s13073-016-0308-x (PMC4855718; doi:10.1186/s13073-016-0308-x)
Supplement: Additional file 11: — Knockdown of endogenous BRD1 using pools of small interfering RNAs (siRNAs). In order to downregulate the endogenous BRD1 level, (A) HEK293T cells were treated with two different pools of four different siRNA directed against BRD1 (BRD1 siRNA) and scrambled siRNA (Ctrl siRNA) as a control. Kits from Dharmacon (Method 1) and Qiagen (Method 2) were used at concentrations of 12.5 and 5.5 nM, respectively, and cells were harvested 24 h after transfection followed by QPCR measurement of BRD1 mRNA. BRD1 mRNA levels were significantly lower (approximately 70–80 % reduction for method 1 and 60 % for method 2) in cells treated with BRD1 directed siRNA compared to cells treated with scrambled siRNA (***P <0.0001). The knockdown experiments showing 80 %, 70 %, and 60 % decreased levels of BRD1 mRNA are referred to as KD1, KD2, and KD3, respectively. Error bars indicate the standard error of the mean (SEM) from n = 3 independent RNA samples. B Expression array data showing log2 fold change values for probes that target BRD1 and seven housekeeping genes: Glyceraldehyde 3-phosphate dehydrogenase (GAPDH), Ubiquitin C (UBC), TATA box binding protein (TBP), hypoxanthine phosphoribosyltransferase 1 (HPRT1), Beta Actin (ACTB), Beta-2 microglobulin (B2M), and glucuronidase beta (GUSB). C Western blot of siRNA silencing of endogenous BRD1 in HEK293T cells using method 1 and 2. BRD1 directed siRNA silencing and control transfections with scrambled siRNA (Ctrl siRNA/Unspecific siRNA) was performed at increasing concentrations. The starting concentrations are approximately 50 % of the manufacturer’s recommended concentrations. D Photometric analysis of western blot in (B), showing decreased levels of BRD1 in cells treated with BRD1 directed siRNA. The BRD1 band intensities are normalized to beta-tubulin intensities and are shown as relative to the highest value. (PDF 165 kb) [file 13073_2016_308_MOESM11_ESM.pdf]

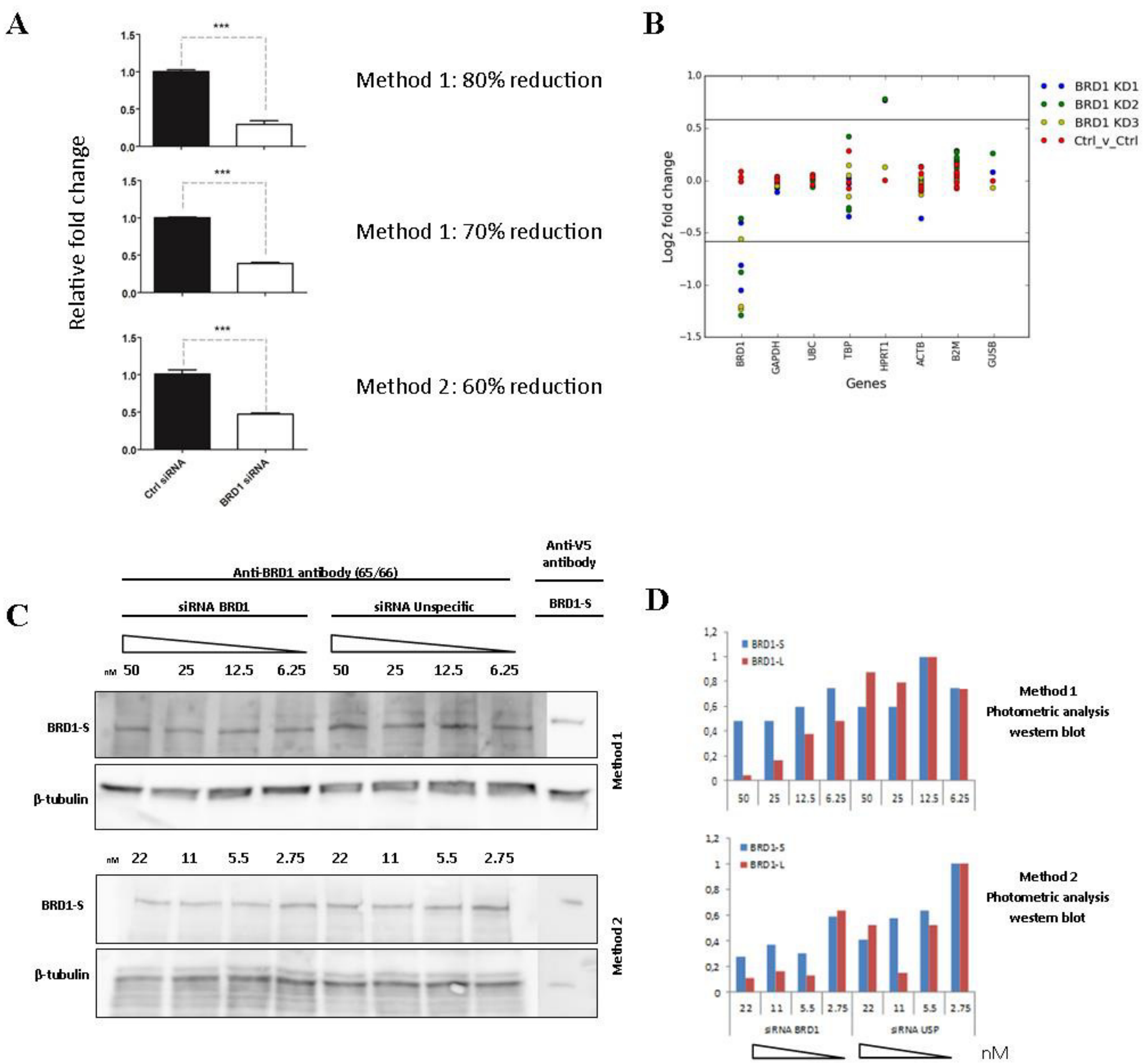
A
Relative fold change
***
Method 1: 80% reduction
Method 1: 70% reduction
Method 2: 60% reduction
Ctrl siRNA
BRD1 siRNA
B
Log2 fold change
Genes
BRD1
GAPDH
UBC
TBP
HPRT1
ACTB
B2M
GUSB
BRD1 KD1
BRD1 KD2
BRD1 KD3
Ctrl_v_Ctrl
C
Anti-BRD1 antibody (65/66)
Anti-V5 antibody
siRNA BRD1
siRNA Unspecific
BRD1-S
nM 50 25 12.5 6.25 50 25 12.5 6.25
nM 22 11 5.5 2.75 22 11 5.5 2.75
β-tubulin
Method 1
Method 2
D
BRD1-S
BRD1-L
Method 1
Photometric analysis
western blot
Method 2
Photometric analysis
western blot
siRNA BRD1
siRNA USP
nM

Knockdown of endogenous BRD1 using pools of small interfering RNAs (siRNAs). In order to down-regulate the endogenous BRD1 level, (A) HEK293T cells were treated with two different pools of 4 different siRNA directed against *BRD1* (BRD1 siRNA) and scrambled siRNA (Ctrl siRNA) as a control. Kits from Dharmacon (Method 1) and Qiagen (Method 2) were used at concentrations of 12.5 and 5.5 nM, respectively, and cells were harvested 24 hours after transfection followed by QPCR measurement of *BRD1* mRNA. *BRD1* mRNA levels were significantly lower (approximately 70 – 80% reduction for method 1 and 60% for method 2) in cells treated with BRD1 directed siRNA compared to cells treated with scrambled siRNA (***$P < 0.0001$). The knockdown experiments showing 80%, 70%, and 60% decreased levels of *BRD1* mRNA are referred to as KD1, KD2, and KD3, respectively. Error bars indicate the standard error of the mean (SEM) from n = 3 independent RNA samples.(B) Expression array data showing $\log_2$ fold change values for probes that target *BRD1* and 7 housekeeping genes: Glyceraldehyde 3-phosphate dehydrogenase (*GAPDH*), Ubiquitin C (*UBC*), TATA box binding protein (*TBP*), hypoxanthine phosphoribosyltransferase 1 (*HPRT1*), Beta Actin (*ACTB*), Beta-2 microglobulin (*B2M*), and glucuronidase beta (*GUSB*). (C) Western blot of siRNA silencing of endogenous BRD1 in HEK293T cells using method 1 and 2. *BRD1* directed siRNA silencing and control transfections with scrambled siRNA (Ctrl siRNA/Unspecific siRNA) was performed at increasing concentrations. The starting concentrations are approximately 50% of the manufactures recommended concentrations. (D) Photometric analysis of western blot in *B*, showing decreased levels of BRD1 in cells treated with *BRD1* directed siRNA. The BRD1 band intensities are normalized to beta-tubulin intensities and are shown as relative to the highest value.
