# Supplementary material for: Identification of the BRD1 interaction network and its impact on mental disorder risk
Source: Genome Med. 2016 May 3;8:53. doi: 10.1186/s13073-016-0308-x (PMC4855718; doi:10.1186/s13073-016-0308-x)
Supplement: Additional file 13: — Integrative analysis of ChIP-seq and gene-expression data. In order to investigate the gene-regulatory potential of BRD1-S and BRD1-L, ChIPseq data were integrated with expression data. A window of +/– 200 kb from BRD1 binding was constructed, fragmented in 9 kb segments, and determined the number of DEGs having TSSs in these segments. As opposed to the 10 kb segment used in the main manuscript the 9 kb segments used here span across 0 on the x-axis. A, B The analysis was performed for DEGs identified after upregulating BRD1-S or BRD1-L or (C, D) downregulating endogenous BRD1. Significantly more up- or downregulated genes were identified in segments where BRD1-S or BRD1-L binds in close proximity to TSSs compared to all segments across the window (P <0.001, 1-sample Student’s t-test). E, F The analysis was also performed after upregulating BRD1-S or BRD1-L and using a narrow window of +/– 5 kb from BRD1 binding sites. In order to investigate the BRD1 gene-regulatory potential while considering all genes on the array (and not only DEGs), probes were segmented according to TSS distance from BRD1 binding site following calculation of the average fold change of all probes within windows +/– 10 kb and +/– 200 kb from BRD1binding sites. F, G These two analyses show the mean expression fold changes combined from several genes and their distance from either BRD1-S or BRD1-L binding on the genome. F The first analysis shows the mean probe fold change in segments of 400 probes and using a +/– 10 kb window. G The second analysis shows the mean probe fold change in segments of 500 probes and using a +/– 200 kb window. (PDF 753 kb) [file 13073_2016_308_MOESM13_ESM.pdf]

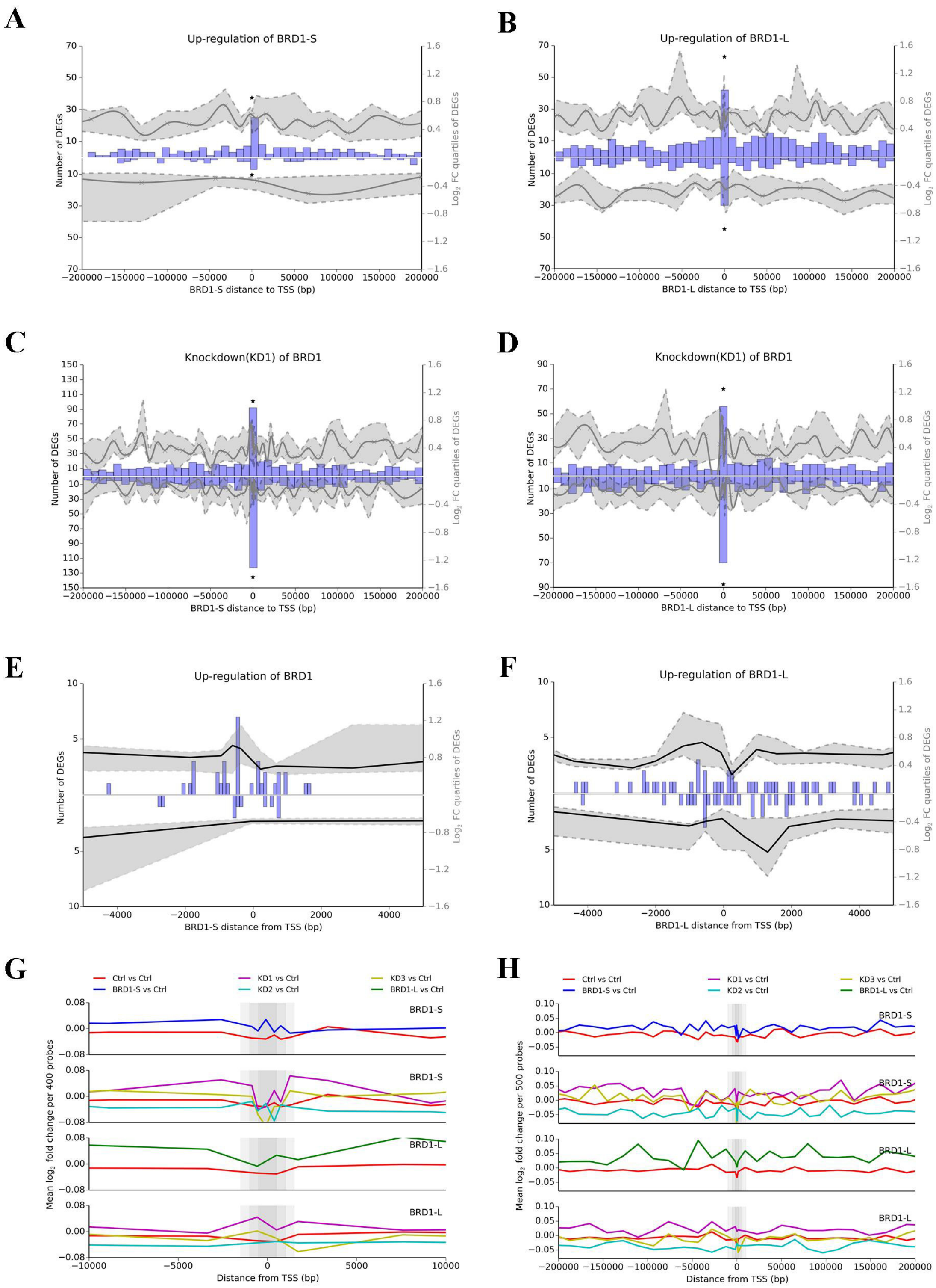

A
Up-regulation of BRD1-S
Number of DEGs
Log2 FC quartiles of DEGs
BRD1-S distance to TSS (bp)
B
Up-regulation of BRD1-L
Number of DEGs
Log2 FC quartiles of DEGs
BRD1-L distance to TSS (bp)
C
Knockdown(KD1) of BRD1
Number of DEGs
Log2 FC quartiles of DEGs
BRD1-S distance to TSS (bp)
D
Knockdown(KD1) of BRD1
Number of DEGs
Log2 FC quartiles of DEGs
BRD1-L distance to TSS (bp)
E
Up-regulation of BRD1
Number of DEGs
Log2 FC quartiles of DEGs
BRD1-S distance from TSS (bp)
F
Up-regulation of BRD1-L
Number of DEGs
Log2 FC quartiles of DEGs
BRD1-L distance from TSS (bp)
G
Ctrl vs Ctrl
BRD1-S vs Ctrl
KD1 vs Ctrl
KD2 vs Ctrl
KD3 vs Ctrl
BRD1-L vs Ctrl
BRD1-S
BRD1-S
BRD1-L
BRD1-L
Mean log2 fold change per 400 probes
Distance from TSS (bp)
H
Ctrl vs Ctrl
BRD1-S vs Ctrl
KD1 vs Ctrl
KD2 vs Ctrl
KD3 vs Ctrl
BRD1-L vs Ctrl
BRD1-S
BRD1-S
BRD1-L
BRD1-L
Mean log2 fold change per 500 probes
Distance from TSS (bp)

Integrative analysis of ChIP-seq and gene-expression data. In order to investigate the gene-regulatory potential of BRD1-S and BRD1-L ChIPseq data was integrated with expression data. A window of +/- 200 kb from BRD1 binding was constructed, fragmented in 9 kb segments, and determined the number of DEGs having TSSs in these segments. As opposed to the 10kb segment used in the main manuscript the 9 kb segments used here span across 0 on the x-axis. (A,B) The analysis was performed for DEGs identified after up-regulating BRD1-S or BRD1-L or (C,D) down-regulating endogenous BRD1. Significantly more up- or down regulated genes were identified in segments where BRD1-S or BRD1-L binds in close proximity to TSSs compared to all segments across the window ($P < 0.001$, 1-sample student's t-test). (E,F) The analysis was also performed after up-regulating BRD1-S or BRD1-L and using a narrow window of +/- 5 kb from BRD1 binding sites. In order to investigate the BRD1 gene-regulatory potential while considering all genes on the array (and not only DEGs), probes were segmented according to TSS distance from BRD1 binding site following calculation of the average fold change of all probes within windows +/- 10kb and +/-200kb from BRD1binding sites. (F,G) These two analyses show the mean expression fold changes combined from several genes and their distance from either BRD1-S or BRD1-L binding on the genome. (F) The first analysis shows the mean probe fold change in segments of 400 probes and using a +/- 10kb window. (G) The second analysis shows the mean probe fold change in segments of 500 probes and using a +/- 200kb window.
