# Supplementary material for: Identification of the BRD1 interaction network and its impact on mental disorder risk
Source: Genome Med. 2016 May 3;8:53. doi: 10.1186/s13073-016-0308-x (PMC4855718; doi:10.1186/s13073-016-0308-x)
Supplement: Additional file 15: — Spatiotemporal mRNA expression of BRD1 in human brain. A RNA-seq data (obtained from Brainspan; http://www.brainspan.org) showing the temporal expression of BRD1 across 26 brain regions; primary auditory cortex, core (A1C), amygdaloid complex (AMY), cerebellar cortex (CBC), cerebellum (CB), caudal ganglionic eminence (CGE), dorsolateral prefrontal cortex (DFC), dorsal thalamus (DTH), hippocampus (hippocampal formation) (HIP), posteroventral (inferior) parietal cortex (IPC), inferolateral temporal cortex (area TEv) (ITC), lateral ganglionic eminence (LGE), primary motor cortex (area M1) (M1C), primary motor-sensory cortex (samples) (M1C-S1C), mediodorsal nucleus of thalamus (MD), anterior (rostral) cingulate (medial prefrontal) cortex (MFC), medial ganglionic eminence (MGE), occipital neocortex (Ocx), orbital frontal cortex (OFC), parietal neocortex (PCx), primary somatosensory cortex (area S1) (S1C), posterior (caudal) superior temporal cortex (area 22c) (STC), striatum (STR), temporal neocortex (TCx), upper (rostral) rhombic lip (URL), primary visual cortex (striate cortex) (V1C), ventrolateral prefrontal cortex (VFC). Age on the x-axis is shown as log10(days) (log10(age)) and the gene expression is shown as the average reads per kilobase per million (avg_rpkm). B Expression microarray data (obtained from the Human Brain Transcriptome, HBT; http://hbatlas.org) showing the temporal expression of BRD1 across six brain regions: neocortex (NCX), hippocampus (HIP), amygdala (AMY), striatum (STR), mediodorsal nucleus of thalamus (MD), cerebellar cortex (CBC). Both datasets show high expression of BRD1 in early fetal stages (until approximately day 180) and both datasets show high expression of BRD1 in the cerebellar cortex throughout the timeline of the datasets. (PDF 208 kb) [file 13073_2016_308_MOESM15_ESM.pdf]

A

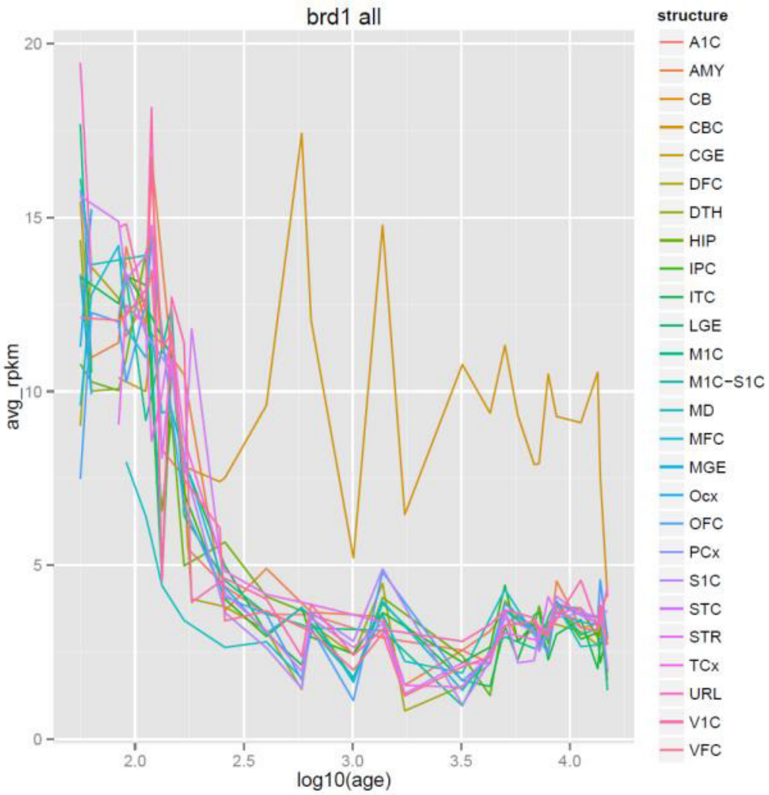
brd1 all
structure
A1C
AMY
CB
CBC
CGE
DFC
DTH
HIP
IPC
ITC
LGE
M1C
M1C-S1C
MD
MFC
MGE
Ocx
OFC
PCx
S1C
STC
STR
TCx
URL
V1C
VFC
avg_rpkm
log10(age)

B

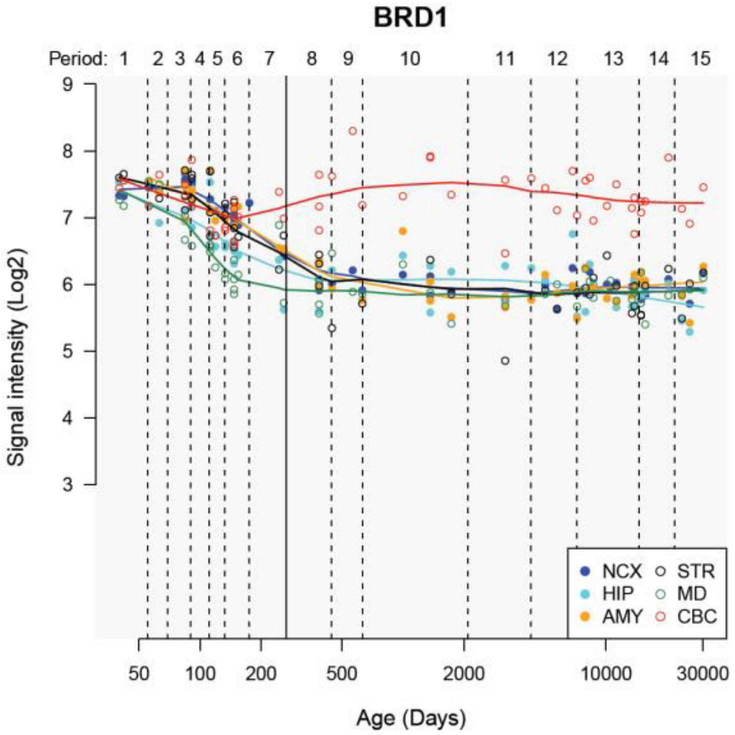
BRD1
Period: 1 2 3 4 5 6 7 8 9 10 11 12 13 14 15
Signal intensity (Log2)
Age (Days)
NCX
HIP
AMY
STR
MD
CBC

Spatiotemporal mRNA expression of *BRD1* in human brain. (A) RNA-seq data (obtained from Brainspan; www.brainspan.org.) showing the temporal expression of *BRD1* across 26 brain regions; A1C: primary auditory cortex (core), AMY: amygdaloid complex, CBC: cerebellar cortex, CB: cerebellum, CGE: caudal ganglionic eminence, DFC: dorsolateral prefrontal cortex, DTH: dorsal thalamus, HIP: hippocampus (hippocampal formation), IPC: posteroventral (inferior) parietal cortex, ITC: inferolateral temporal cortex (area TEv), LGE: lateral ganglionic eminence, M1C: primary motor cortex (area M1), M1C-S1C: primary motor-sensory cortex (samples), MD: mediodorsal nucleus of thalamus, MFC: anterior (rostral) cingulate (medial prefrontal) cortex, MGE: medial ganglionic eminence, Ocx: occipital neocortex, OFC: orbital frontal cortex, PCx: parietal neocortex, S1C: primary somatosensory cortex (area S1), STC: posterior (caudal) superior temporal cortex (area 22c), STR: striatum, TCx: temporal neocortex, URL: upper (rostral) rhombic lip, V1C: primary visual cortex (striate cortex), VFC: ventrolateral prefrontal cortex. Age on the x-axis is shown as $\log_{10}$(days) (log10(age)) and the gene expression is shown as the average reads per kilobase per million (avg_rpkm). (B) Expression microarray data (obtained from the Human Brain Transcriptome, HBT; http://hbatlas.org/) showing the temporal expression of *BRD1* across 6 brain regions; NCX: neocortex, HIP: hippocampus, AMY: amygdala, STR: striatum, MD: mediodorsal nucleus of thalamus, CBC: cerebellar cortex. Both datasets show high expression of *BRD1* in early fetal stages (until approximately day 180) and both datasets show high expression of *BRD1* in the cerebellar cortex throughout the timeline of the datasets.
