# Supplementary material for: Identification of the BRD1 interaction network and its impact on mental disorder risk
Source: Genome Med. 2016 May 3;8:53. doi: 10.1186/s13073-016-0308-x (PMC4855718; doi:10.1186/s13073-016-0308-x)
Supplement: Additional file 17: — Spatiotemporal co-expression landscapes of BRD1-S and BRD1-L PPIs and PTGs in the human brain. The fraction of co-expressed genes with BRD1 across 32 spatiotemporal intervals. A The fraction of genes co-expressed with BRD1 across all genes (gray) and genes in the BRD1-S and BRD1-L PPI networks (red and orange). None of these were significant at P <0.05 after adjusting for multiple tests. (#) denotes P <0.05 before correcting for multiple testing. B The fraction of genes co-expressed with BRD1 across all genes (gray) and genes in the BRD1-S and BRD1-L PTGs + PPI networks (red and orange). A one-sided binominal test was performed for each BRD1 sub-network and spatiotemporal interval compared to the expected fraction in the background where the asterisk (*) denotes P <0.05 after adjusting for multiple tests. (PDF 322 kb) [file 13073_2016_308_MOESM17_ESM.pdf]

A

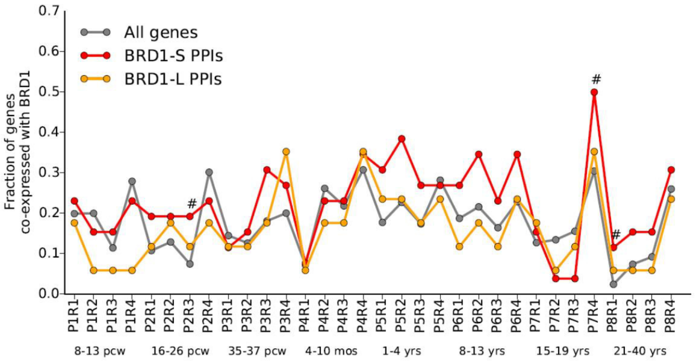

B

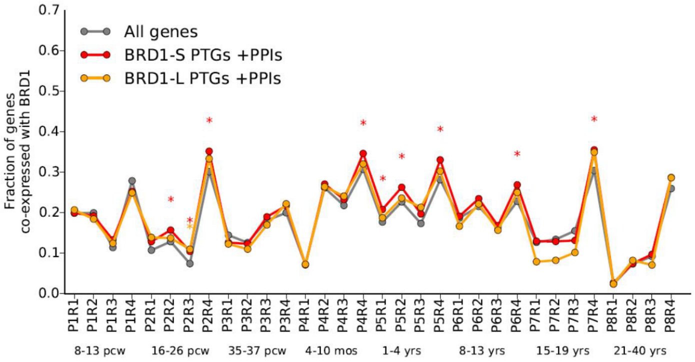

Spatiotemporal co-expression landscapes of BRD1-S and BRD1-L PPIs and PTGs in human brain. The fraction of co-expressed genes with BRD1 across 32 spatiotemporal intervals. (A) The fraction of genes co-expressed with BRD1 across all genes (grey) and genes in the BRD1-S and BRD1-L PPI networks (red and orange). None of these were significant at $P < 0.05$ after adjusting for multiple tests. (#) denote $P < 0.05$ before correcting for multiple testing. (B) The fraction of genes co-expressed with BRD1 across all genes (grey) and genes in the BRD1-S and BRD1-L PTGs + PPI networks (red and orange). A one-sided binominal test was performed for each BRD1 sub-network and spatiotemporal interval compared to the expected fraction in the background where the asterisk (*) denote $P < 0.05$ after adjusting the P-value for multiple tests.
